# Prognostic and immunological role of SERPINH1 in pan-cancer

**DOI:** 10.3389/fgene.2022.900495

**Published:** 2022-08-29

**Authors:** Huage Zhong, Zheng Wang, Xiaoxia Wei, Yaning Liu, Xiaoliang Huang, Xianwei Mo, Weizhong Tang

**Affiliations:** ^1^ Division of Colorectal and Anal Surgery, Department of Gastrointestinal Surgery, Guangxi Medical University Cancer Hospital, Nanning, China; ^2^ Guangxi Clinical Research Center for Colorectal Cancer, Nanning, China; ^3^ Centre of Imaging Diagnosis, Affiliated Tumor Hospital of Guangxi Medical University, Nanning, China

**Keywords:** SERPINH1, pan-cancer, immune checkpoints, immunotherapy, prognosis

## Abstract

**Background:** The SERPINH1 gene plays a vital part in tumorigenesis and development, whereas its potential as an immunotherapy target is still unknown. Hence, this research aimed to probe the roles of SERPINH1 in human tumors.

**Method:** Using The Cancer Genome Atlas (TCGA), Genotype-Tissue Expression (GTEx) database, Oncomine, and SangerBox software, the pan-cancer expression of SERPINH1 and its correlation were systematically analyzed. SERPINH1 protein information was detected by the Human Protein Atlas (HPA) database and STRING database. The genomic alterations of SERPINH1 were studied using the c-BioPortal database. The influence of SERPINH1 on prognosis was analyzed using Kaplan–Meier plotter. The R package “clusterProfiler” was used for enrichment analysis to detect the role of SERPINH1. The TIMER2 database was used to further analyze the correlation between the immune cell infiltration score of TCGA samples and the expression of SERPINH1.

**Results:** SERPINH1 overexpression was related to worse survival status in pan-cancer. In addition, high expression of SERPINH1 was positively associated with tumor stage and poor prognosis. Moreover, SERPINH1 played an important role in tumor microenvironment and immune regulation. Our study revealed that SERPINH1 expression has a strong correlation with immune cell filtration, immune regulation, chemokines, and immune checkpoints.

**Conclusion:** Our research found that SERPINH1 was a risk factor and predictor of poor prognosis in various tumors. High expression of SERPINH1 may contribute to tumor immune-suppressive status. Also, SERPINH1 may become a potential immunotherapy target in pan-cancer.

## Introduction

According to the report of Global Cancer Statistics 2020 published by IARC, 19.3 million new cases and 10 million cancer deaths occurred worldwide in 2020 (Sung et al., 2021). As the second most frequent cause of death in the world, cancer is a serious threat to people’s life safety. In consequence, it is urgent for us to conduct in-depth research to clarify the pathogenesis of tumor development.

Pan-cancer analysis of a gene is conducive to evaluate its relevance to clinical prognosis and potential molecular mechanisms. As bioinformatics analysis has been widely used to screen and analyze genes associated with various disease progression, a number of gene profiles and large clinical databases of diverse tumors can be obtained based on public databases such as TCGA and GTEx (Benyamin et al., 2014; Chen et al., 2021). With these large numbers of collections, we can perform pan-cancer expression analysis.

Serpin peptidase inhibitor branch H member 1 (SERPINH1) belongs to the serpin superfamily, is located at 11q13.5, and encodes heat shock protein 47 (HSP47) (Wu et al., 2019). SERPINH1 has been identified as a key chaperone protein that regulates and maintains cell protease homeostasis (Hirayoshi et al., 1991), and is closely correlated with the occurrence and development of tumors. Previous research studies have shown that SERPINH1 is aberrantly expressed in a variety of cancers, for example, the expression of SERPINH1 is significantly increased in colorectal cancer cells (Mori et al., 2017). The high expression of SERPINH1 in some tumors is related to cell proliferation, tumor stage, pathological grade, and poor prognosis (Lee et al., 2016; Yamada et al., 2018). However, human pan-cancer evidence regarding the potential effects of the SERPINH1 gene in various tumor types is incompletely understood. We believe that SERPINH1 is likely to be a new target for tumor therapy, so it is very urgent to carry out pan-cancer research on SERPINH1.

SERPINH1 is a crucial regulator of collagen biosynthesis and its structural assembly and has been designated as a potential biomarker or therapeutic target for many diseases (Kumar et al., 2017; Qi et al., 2018; Fan et al., 2020; Tian et al., 2020; Wang et al., 2022). Studies have confirmed that SERPINH1 may promote cancer growth and invasion by regulating the extracellular matrix network (Zhu et al., 2015). SERPINH1 is related to tumor immunity and can change the composition of immune cells by inducing the tumor microenvironment (Li et al., 2020), but its mechanism is still unclear and needs further study.

Our research is the first pan-cancer analysis of SERPINH1 using the TCGA project and GTEx database. We also carried out gene expression, gene changes, survival status, immune infiltration, and functional enrichment analysis to investigate the potential molecular mechanisms of SERPINH1 in the pathogenesis or clinical prognosis of different cancers. Our results suggest that SERPINH1 expression affects the prognosis of patients with pan-cancer and is significantly associated with tumor immune invasion. In addition, it would serve as a potential target for cancer immunotherapy.

## Materials and methods

### Data collection

The UCSC Xena database (https://xenabrowser.net/datapages/) was used to download The Cancer Genome Atlas (TCGA) Pan-Cancer and the Genotype-Tissue Expression (GTEx) RNA expression and clinical data. The DNA copy number was downloaded from the c-BioPortal database (https://www.cbioportal.org/). To verify the predictive value of SERPINH1 in immunotherapy response, gene expression data and clinical data of GSE78220 and IMvigor210 were downloaded from the Gene Expression Omnibus (GEO) database. Also, the PPI network of SERPINH1 was downloaded from the STRING database.

### Single-cell RNA-sequencing data collection and data analysis

Single-cell RNA-sequencing data derived from two KIRC patients who underwent radical nephrectomy were obtained from online repositories of GEO (GSE152938). All samples were sequenced using HiSeq X10 (Illumina, San Diego, CA) with set standard parameters (Foox et al., 2021). Preliminary sequencing files (.bcl) were converted to FASTQ files based on CellRanger (version 3.0.2) R (version 3.5.2), and the Seurat R package (version 3.1.1) was used for QC and secondary analysis.

### Protein-level analysis

The Human Protein Atlas (HPA: https://www.proteinatlas.org/) database was used to detect human tumors and normal tissues in SERPINH1 protein levels. The protein–protein interaction network (PPI) of SERPINH1 was constructed using the STRING (https://string-db.org/) database.

### Differential expression analysis of SERPINH1

Using the Oncomine database, the largest oncogene chip database and integrated data mining tools, the differentiation of SERPINH1 expression levels in diverse types of tumors and normal tissues was revealed (Rhodes et al., 2007). Using SangerBox software and based on the TCGA database, the differences of SERPINH1 expression levels between cancer and adjacent tissues were compared.

### Genetic alteration analysis

The genetic alteration analysis of SERPINH1 was searched in the c-BioPortal web (https://www.cbioportal.org/). The “Quick select” and “TCGA Pan-Cancer Atlas Studies” options were selected. The frequency of genetic changes, copy number changes (CNA), and mutation types were described in the “Cancer Type Summary” module of the entire TCGA database.

### Pathway exploration of SERPINH1 at the single-cell level

CancerSEA contains 41,900 tumor cells from 25 cancers with 14 cancer-associated functional states, providing a map of the functional states of single cells in cancer and linking these functional states to protein-coding genes at the single-cell level to facilitate mechanistic understanding of functional differences in cancer cells (Yuan et al., 2019). We used the CancerSEA database to explore the correlation between SERPINH1 expression and functional states in various cancers.

### Survival analysis

We applied Kaplan–Meier analysis to show the disparity in survival between patients with high- and low-expression groups to assess the prognostic value of SERPINH1. The R packages used in these operations are survival, glmnet, survival ROC, and survminer. Cox regression analysis was used to detect the correlation between the expression of SERPINH1 in the R environment and overall survival (OS), disease-free survival (DFS), disease-specific survival (DSS), and progression-free survival (PFS).

### Immune infiltration analysis

The association between SERPINH1 expression and immune infiltration was found using the TIMER2 database and “CIBERSOFT” method. We analyzed the expression of SERPINH1 in B cells, CD4^+^ T cells, CD8^+^ T cells, neutrophils, macrophages, dendritic cells (DC), and so on. In addition, the correlation between SERPINH1 expression and Immune score, Stromal Score, and ESTIMATE Score was investigated using ESTIMATE.

### Gene set enrichment analyses

Using the “clusterProfiler” R package, the pathways affected by SERPINH1 were identified through gene set enrichment analysis (GSEA). The sorted gene list obtained from the fold-change of the average gene expression between patients with high- and low-SERPINH1 expression represents the input file. The Kyoto Encyclopedia of Genes and Genomes (KEGG) pathways and HALLMARK pathways were used to evaluate biological processes.

### Statistical analysis

The TPM SEIPINH1 mRNA expression value was used to establish SERPINH1 low- and high-expression groups. The Spearman correlation test was used to evaluate the correlation between the expression of SERPINH1 and the target of interest. We used COX regression analysis to calculate HRs and log-rank P-values in the survival analysis. The P-value was less than 0.05 as the difference was significant.

## Result

### SERPINH1 expression analysis in pan-cancer

**FIGURE 1:**
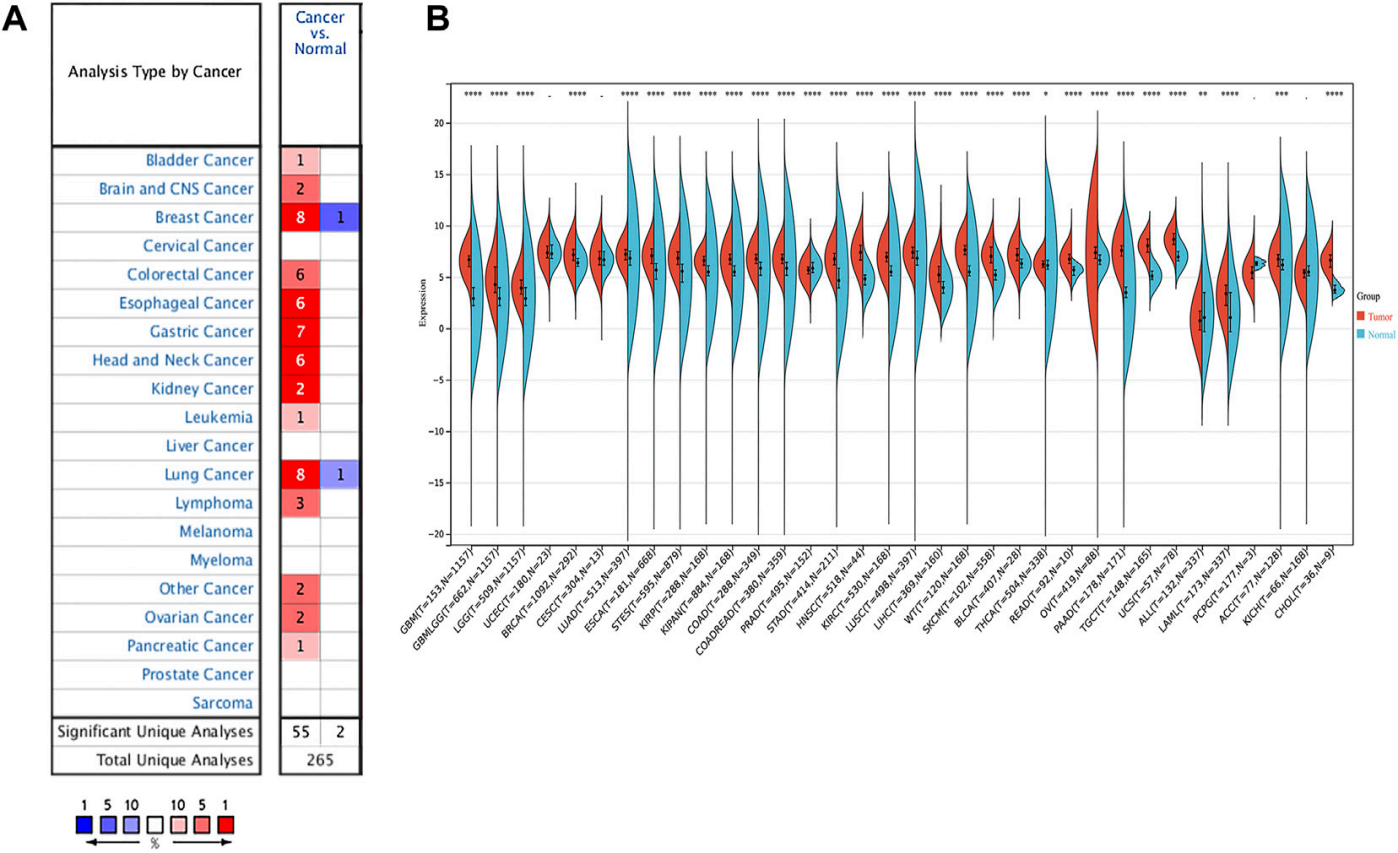
SERPINH1 expression analysis in pan-cancer. **(A)** Expression of SERPINH1 in different cancers and matched normal tissues in Oncomine. The number in each cell is the number of datasets. The red (blue) 1% represents the top 1% of genes that are highly (lowly) expressed in the tumor in the GEO database. Blanks represent data sets that are not in the top 10% of high or low tumor expression. **(B)** Expression of SERPINH1 in different cancer types in the GTEx and TCGA database.

The RNA expression of SERPINH1 in pan-cancer data was first evaluated using the Oncomine database. The results revealed that SERPINH1 mRNA level was significantly highly expressed in bladder, brain and CNS, breast, colorectal, esophageal, gastric, head and neck, leukemia, lung, lymphoma, ovarian, pancreatic, and other cancers in comparison to corresponding normal tissues (Figure 1A). The GTEx database was used to further explore the mRNA expression of SERPINH1 in pan-cancer of TCGA. The result suggested that SERPINH1 was overexpressed in GBM (glioblastoma multiforme), GBMLGG (glioma), LGG (brain lower grade glioma), BRCA (breast invasive carcinoma), LUAD (lung adenocarcinoma), ESCA (esophageal carcinoma), STES (stomach and esophageal carcinoma), KIRP (kidney renal papillary cell carcinoma), KIPAN [Pan-kidney cohort (KICH + KIRC + KIRP)], COAD (colon adenocarcinoma), COADREAD (colon adenocarcinoma/rectum adenocarcinoma esophageal carcinoma), STAD (stomach adenocarcinoma), HNSC (head and neck squamous cell carcinoma), KIRC (kidney renal clear cell carcinoma), LUSC (lung squamous cell carcinoma), LIHC (liver hepatocellular carcinoma), WT (high-risk Wilms tumor), SKCM (skin cutaneous melanoma), BLCA (bladder urothelial carcinoma), READ (rectum adenocarcinoma), OV (ovarian serous cystadenocarcinoma), PADD (pancreatic adenocarcinoma), TGCT (testicular germ cell tumors), UCS (uterine carcinosarcoma), LAML (acute myeloid leukemia), ACC (adrenocortical carcinoma), and CHOL (cholangiocarcinoma). Meanwhile, the underexpression of SERPINH1 was found only in PRAD (prostate adenocarcinoma) (Figure 1B). The abbreviations and full name of these cancers can be found in Supplementary Table S1. The abovementioned results indicate that SERPINH1 was overexpressed in most human tumors and promotes their development.

### Relationship between SERPINH1 expression and cancer stage

**FIGURE 2:**
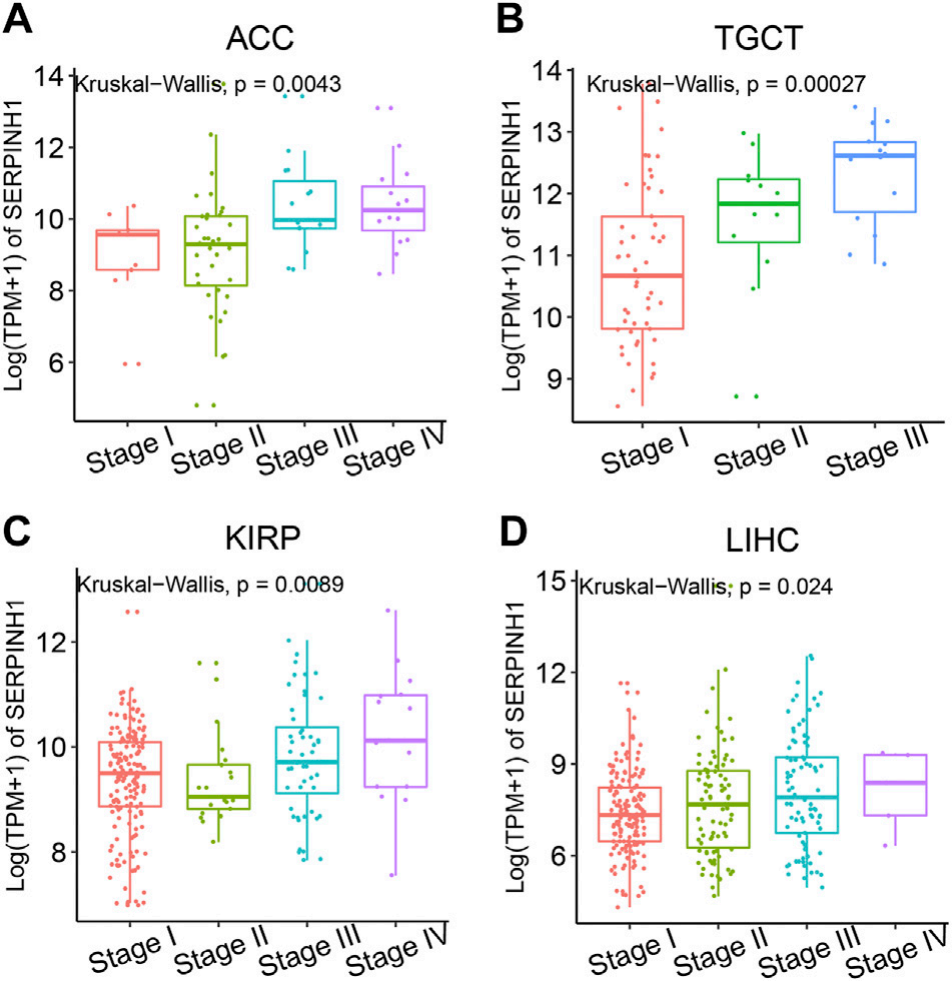
Relationship between SERPINH1 expression and cancer stage. SERPINH1 expression was analyzed by main pathological stages (stage I, stage II, stage III, and stage IV) of ACC **(A)**, TGCT **(B)**, KIRP **(C)**, and LIHC **(D)**. Log (TPM + 1) was used for the log scale.

To better realize the relationship between the expression of SERPINH1 and tumor stages, we further analyzed SERPINH1 expression in diverse World Health Organization tumor stages. The results revealed that SERPINH1 was highly expressed in the advanced stage of ACC, TGCT, KIRP, and LIHC (Figures 2A–D). Particularly in ACC and TGCT, the expression of SERPINH1 increased with the progression of tumor stage. It suggested that SERPINH1 was closely related to this tumor progression. The expression of SERPINH1 in different tumors at different stages is shown in Supplementary Figure S1. In order to clarify the relationship between SERPINH1 expression and early and late stages of tumor, we combined stages I–II as early and III–IV as late for analysis. We found that SERPINH1 overexpressed in later stages in most cancers, including ACC, BLCA, KIRC, LIHC, and KIRP (Supplementary Figure S2, all *p* < 0.05). On the contrary, SEIPINH1 expression in the early stage was only found in KICH. But it had no statistical significance. SERPINH1 expression tends to increase in patients with stage III–IV tumor, including BRCA, COAD, HNSC, CHOL, ESCA, LUSC, PAAD, LUAD, MESO, READ, SKCM, TGCT, UVM, STAD, and THCA. However, the difference is not statistically significant (Supplementary Figure S2). In addition, the mean values and fold-changes of SERPINH1 expression in different early and advanced stages are shown in Table 1. The results revealed that SERPINH1 expression had a positive correlation with tumor stage in most of the cancers.

**TABLE 1:** Mean expression and fold-change of SERPINH1 in different early and advanced tumors.

Type	Expression mean in early stage	Expression mean in advanced stage	Fold-change	p-value
ACC	9.087282915	10.36767862	0.876501216	0.000217458
BLCA	10.12690295	10.45591801	0.968533126	0.015647385
BRCA	10.25907595	10.34460248	0.991732256	0.341418718
CHOL	9.345009284	9.944059695	0.939757963	0.361526902
COAD	9.618512171	9.847812636	0.976715594	0.093334244
ESCA	10.05939074	10.1827528	0.987885195	0.637603076
HNSC	10.64936933	10.60821109	1.003879847	0.800383083
KICH	7.672732749	8.119905195	0.944928859	0.346660863
KIRC	9.875601272	10.18265253	0.969845651	0.002886189
KIRP	9.380656256	9.923894979	0.945259525	0.001027745
LIHC	7.501038702	8.12568555	0.923126874	0.004090039
LUAD	10.32709135	10.50281462	0.983268936	0.165713842
LUSC	10.68983599	10.63927839	1.004751976	0.719149067
MESO	12.35671548	12.54487081	0.985001413	0.516348945
PAAD	10.73131579	10.95250252	0.979804914	0.679259262
READ	9.730222776	9.703203568	1.002784566	0.915331409
SKCM	10.24335544	10.32035358	0.992539196	0.821295677
STAD	9.761521733	9.782094627	0.997896883	0.871405746
TGCT	10.97720853	12.33111648	0.890203945	0.0000302024
THCA	9.022104483	9.06259806	0.995531792	0.61288199
UVM	9.326413773	9.621338122	0.969346847	0.109765198

### Genetic alterations of SERPINH1

**FIGURE 3:**
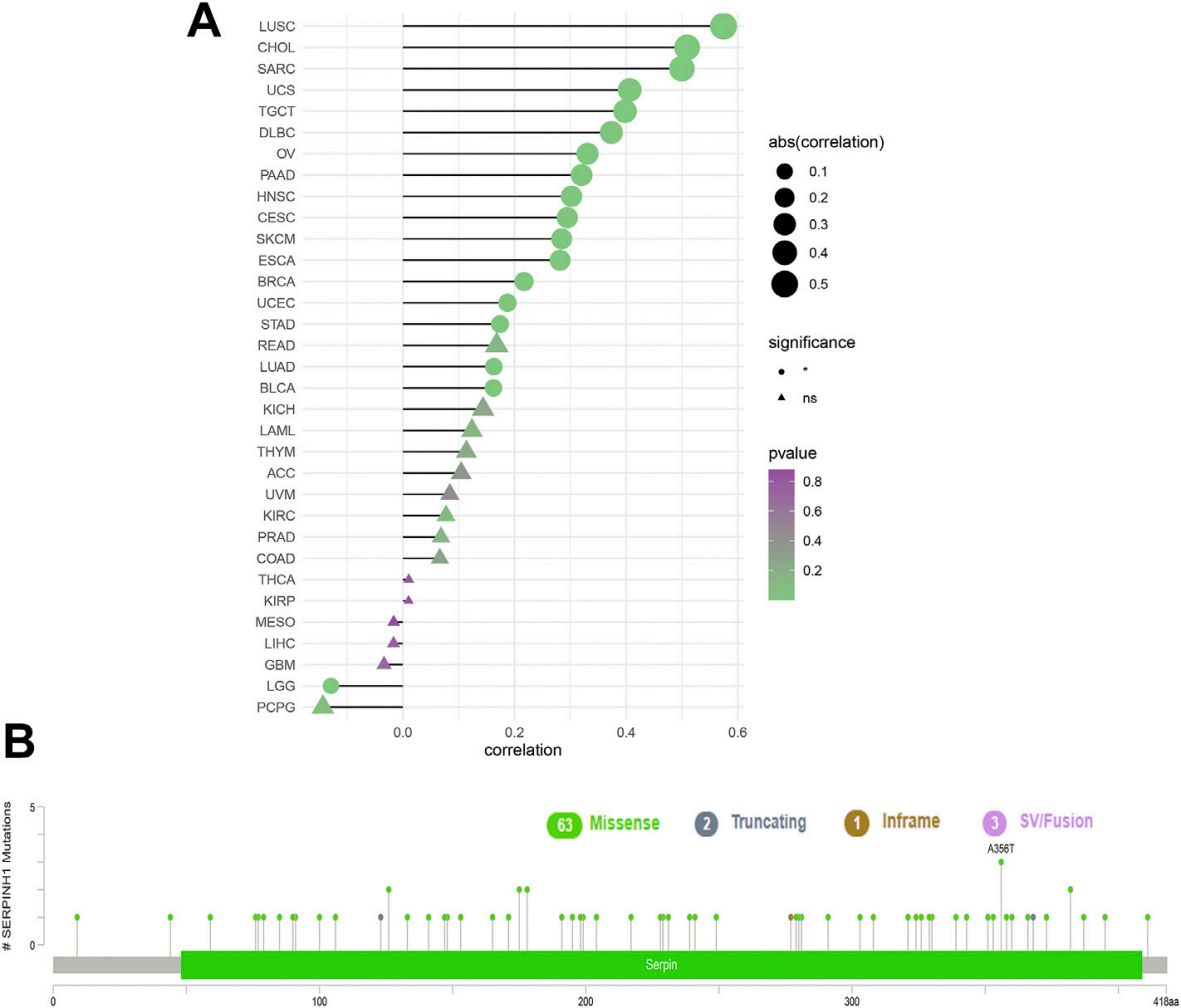
Genetic alterations of SERPINH1. **(A)** Copy number alterations and **(B)** mutation types in SERPINH1 with the most frequency of missense mutations.

To analyze the relevance of the SERPINH1 gene mutation in different kinds of cancers, the cBioPortal tool was used to detect the genetic alterations of SERPINH1 in TCGA. As illustrated in Figure 3A, we found that the amplification type of copy number alteration has a positive correlation in most tumors, especially LUSC, CHOL, and SARC. The types, sites, and case numbers of the SERPINH1 genetic mutation are shown in Figure 3B. It indicated that the main type of genetic alteration of SERPINH1 was missense mutation.

### PPI network and protein level of SERPINH1

**FIGURE 4:**
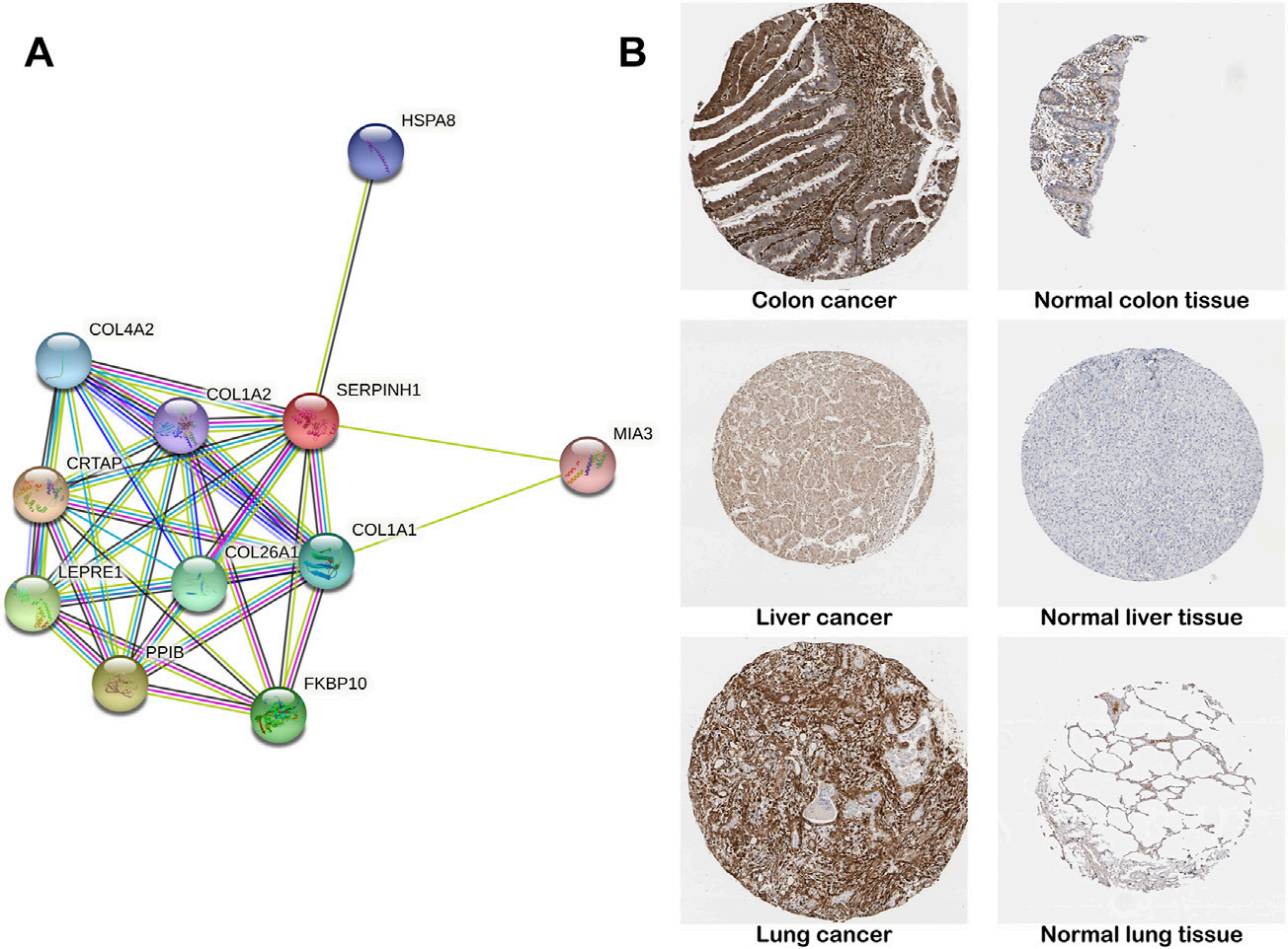
PPI network and protein level of SERPINH1. **(A)** SERPINH1 PPI network. PPI, protein–protein interaction. Each node in the figure represents a protein structure. Violet-red indicates from curated databases, copper indicates experimentally determined. Lime, red, and blue represent gene neighborhood, gene fusions, and gene co-occurrence, respectively. Yellow-green indicates textmining; black indicates co-expression, and navy blue indicates protein homology. **(B)** Representative immunohistochemical staining results of SERPINH1 protein in different tumor tissues and normal tissues.

The construction of PPI network was based on the STRING database, which stored information on 13,808,38,440 interactions for 2,031 species and 9,643,763 proteins (Franceschini et al., 2013). We constructed a PPI network for SERPINH1 to explore SERPINH1 and related proteins. We found that SERPINH1 was integrated with COL1A1, COL1A2, COL4A2, HSPA8, MIA3, CRTAP, LEPRE1, PPIB, and FKBP10. That means SERPINH1 may participate in the regulation of extracellular matrix collagen formation (Figure 4A). We have studied SERPINH1 protein levels using the HPA database. The result showed that the SERPINH1 protein levels were higher in tissues of colon, liver, and lung cancer than in corresponding normal tissues (Figure 4B). It suggested that SERPINH1 may become a potential tumor marker.

### Prognostic significance of SERPINH1

**FIGURE 5:**
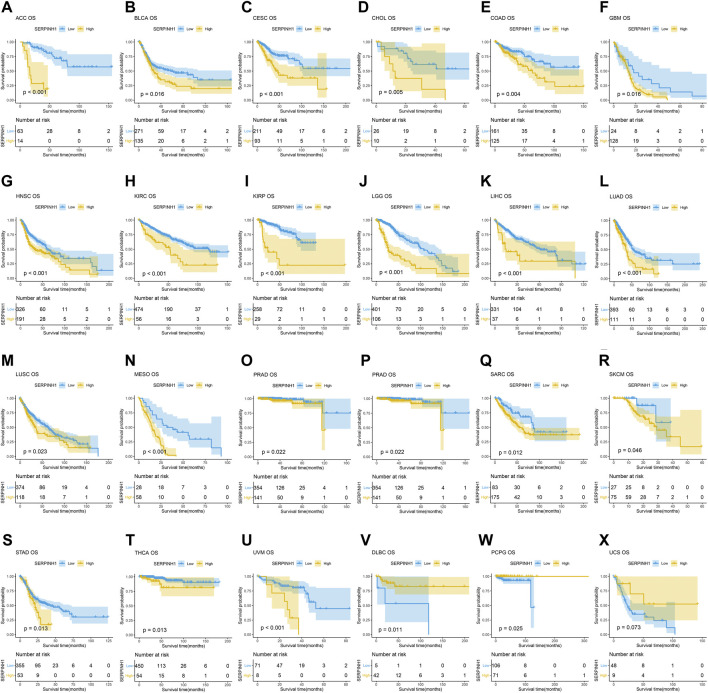
Kaplan–Meier overall survival (OS) analysis. **(A–U)** High expression of SERPINH1 is related to poor OS; **(V–X)** low expression of SERPINH1 is related to poor OS.

**FIGURE 6:**
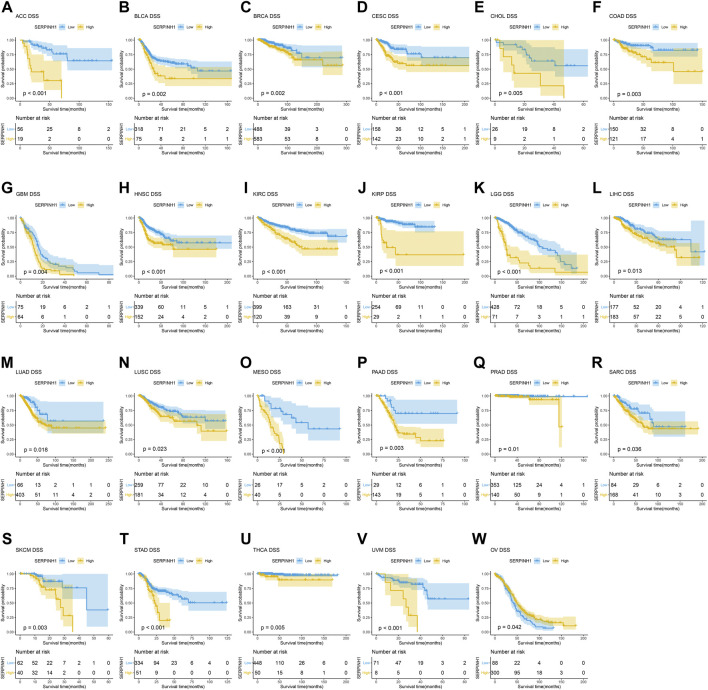
Kaplan–Meier disease-specific survival (DSS) analysis. **(A–V)** High expression of SERPINH1 is related to poor DSS; **(W)** low expression of SERPINH1 is related to poor DSS.

**FIGURE 7:**
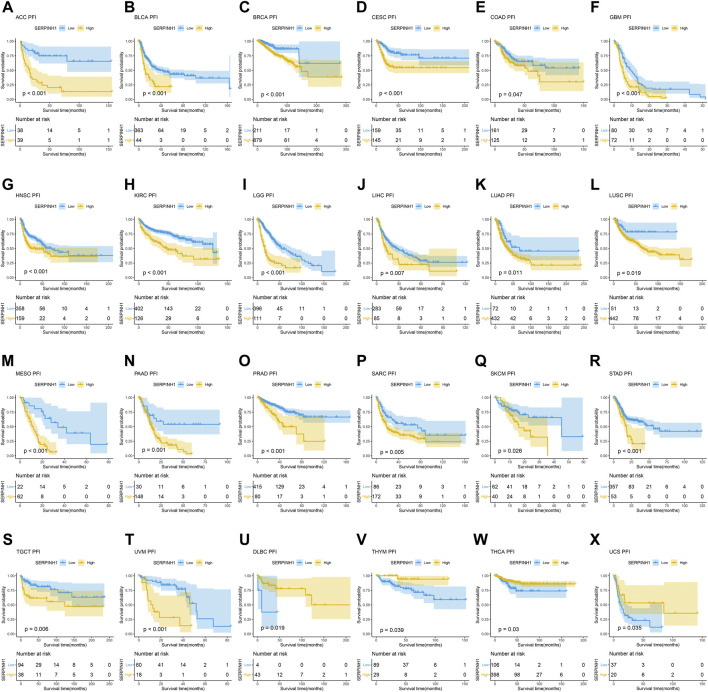
Kaplan–Meier relapse-free survival (PFI) analysis. **(A–T)** High expression of SERPINH1 is related to poor PFI; **(U–X)** low expression of SERPINH1 is related to poor PFI.

In this research, the prognostic significance of SERPINH1 for pan-cancer was evaluated using the Kaplan–Meier analysis and univariate Cox regression analysis. The Kaplan–Meier overall survival (OS) analysis revealed that SERPINH1 was a risk factor for patients with ACC, BLCA, CESC, CHOL, COAD, GBM, HNSC, KIRC, KIRP, LGG, LIHC, LUAD, LUSC, MESO, PAAD, SARC, SKCM, STAD, THCA, UVM, DLBC, PCPG, and USC (Figure 5). For disease-specific survival (DSS), the result revealed that high SERPINH1 expression was related to patients with ACC, BLCA, BRCA, CESC, CHOL, COAD, GBM, HNSC, KIRC, KIRP, LGG, LIHC, LUAD, LUSC, MESO, PAAD, PRAD, and SARC (Figure 6). Patients with high SERPINH1 expression were significantly less in PFI, including ACC, BLCA, BRCA, CESC, COAD, GBM, HNSC, KIRC, LGG, LIHC, LUAD, LUSC, MESO, PAAD, PRAD, SARC, SKCM, STAD, TGCT, UVM, DLBC, THYM, THCA, and UCS (Figure 7). Supplementary Figure S3 shows the results of the analysis of survival curves where the difference was not statistically significant. For visual and clarity reasons, the hazard ratio, 95% CI, and *p*-value for OS, PFI, and DSS are shown in the Supplementary Table S2.

**FIGURE 8:**
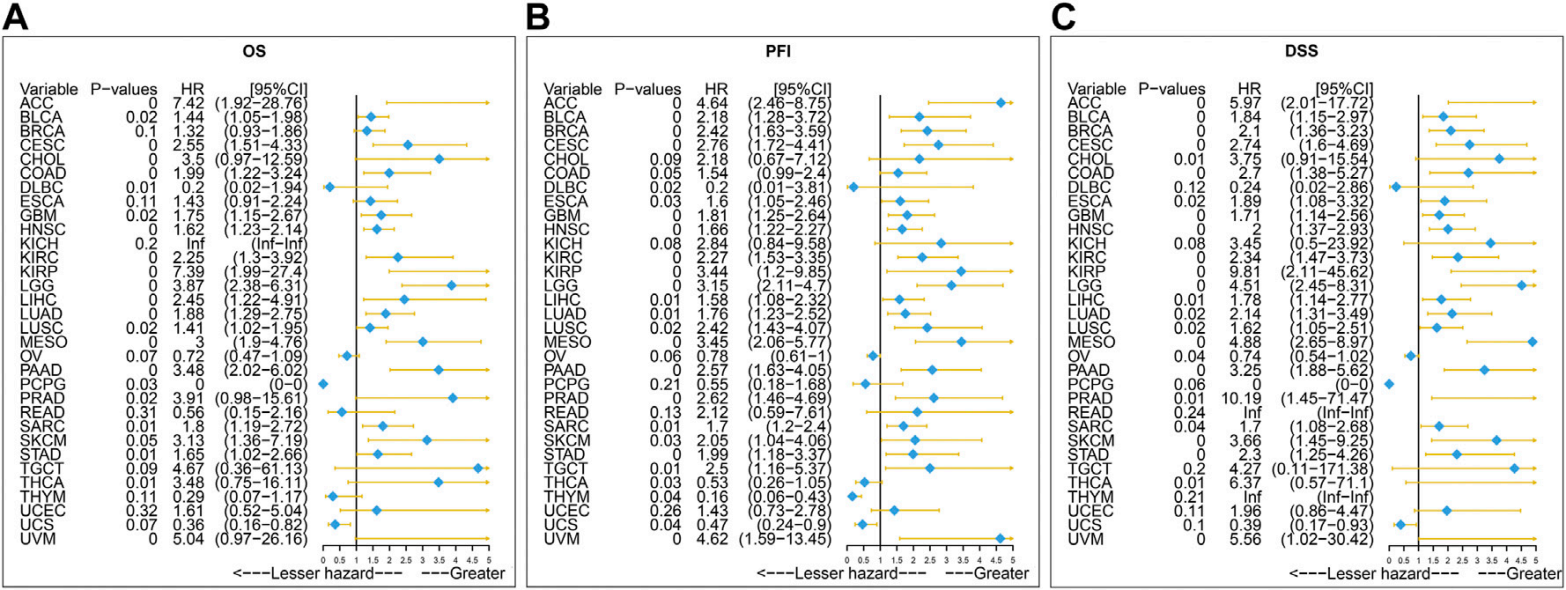
Univariate Cox regression analysis of SERPINH1. These forest maps, respectively, reveal the univariate Cox regression results of SERPINH1 on OS **(A)**, PFI **(B),** and DSS **(C)** in TCGA pan-cancer. Items with a hazard ratio greater than 1 showed that the SERPINH1 expression was a contributing factor to death.

Furthermore, the result of univariate Cox regression analysis found that high expression of SERPINH1 was a harmful factor of OS in ACC, BLCA, CESC, COAD, GBM, HNSC, KICH, KIRC, LGG, LIHC, LUSC, MESO, PAAD, SARC, SKCM, and STAD. However, it was found protective in CHOL, OV, and THCA (Figure 8A). For DSS, overexpression of SERPINH1 was associated with the decrease of DSS in ACC, BLCA, BRCA, CESC, COAD, ESCA, GBM, HNSC, KIRC, KIRP, LGG, LIHC, LUAD, MESO, PAAD, SARC, SKCM, STAD, and UVM; however, it increased the DSS in CHOL, OV, and THCA (Figure 8B). Meanwhile, the result of PFI also revealed that higher expression of SERPINH1 was concerned with worse PFI in ACC, BLCA, BRCA, CESC, ESCA, GBM, HNSC, KIRC, KIRP, LGG, LIHC, LUAD, LUSC, MESO, PAAD, PRAD, SARC, SKCM, STAD, TGCT, and UVM while better PFI in DLBC, THCA, THYM, and UCS (Figure 8C).

### GSEA of SERPINH1

**FIGURE 9:**
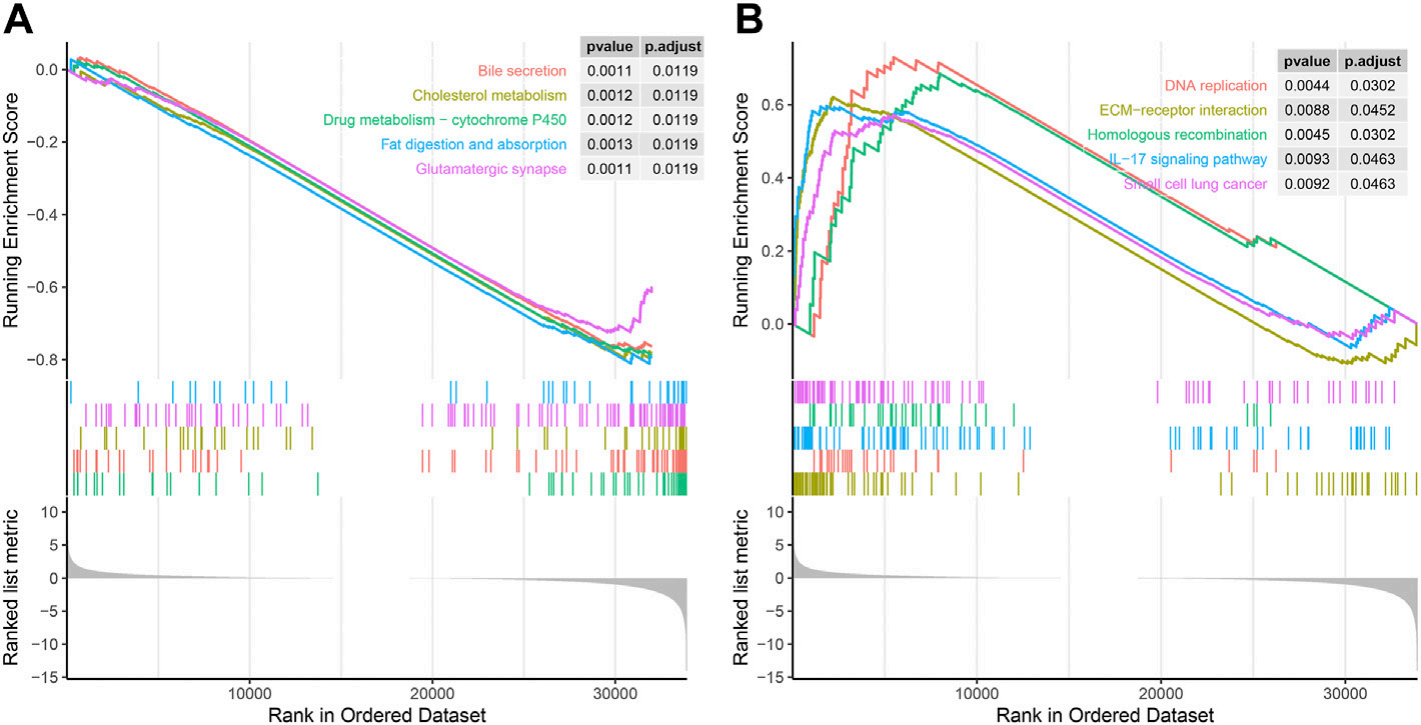
GSEA of SERPINH1. **(A)** Enrichment analysis in the high SERPINH1 expression group. **(B)** Enrichment analysis in the low SERPINH1 expression group.

Next, we used GSEA to explore the KEGG pathway of SERPINH1 expression. As we can see in Figure 9A, low expression of SERPINH1 was significantly associated with bile secretion, cholesterol metabolism, drug metabolism-P450, fat digestion and absorption, and glutamatergic synapse. However, high expression of SERPINH1 was mainly correlated with DNA replication, ECM–receptor interaction, homologous recombination, IL-17 signaling pathway, and small cell lung cancer (Figure 9B). The results suggested that the high expression of SERPINH1 affected the occurrence and development of tumors through the abovementioned pathways and was related to the malignant progression of tumors.

### Immune cell infiltration analysis

**FIGURE 10:**
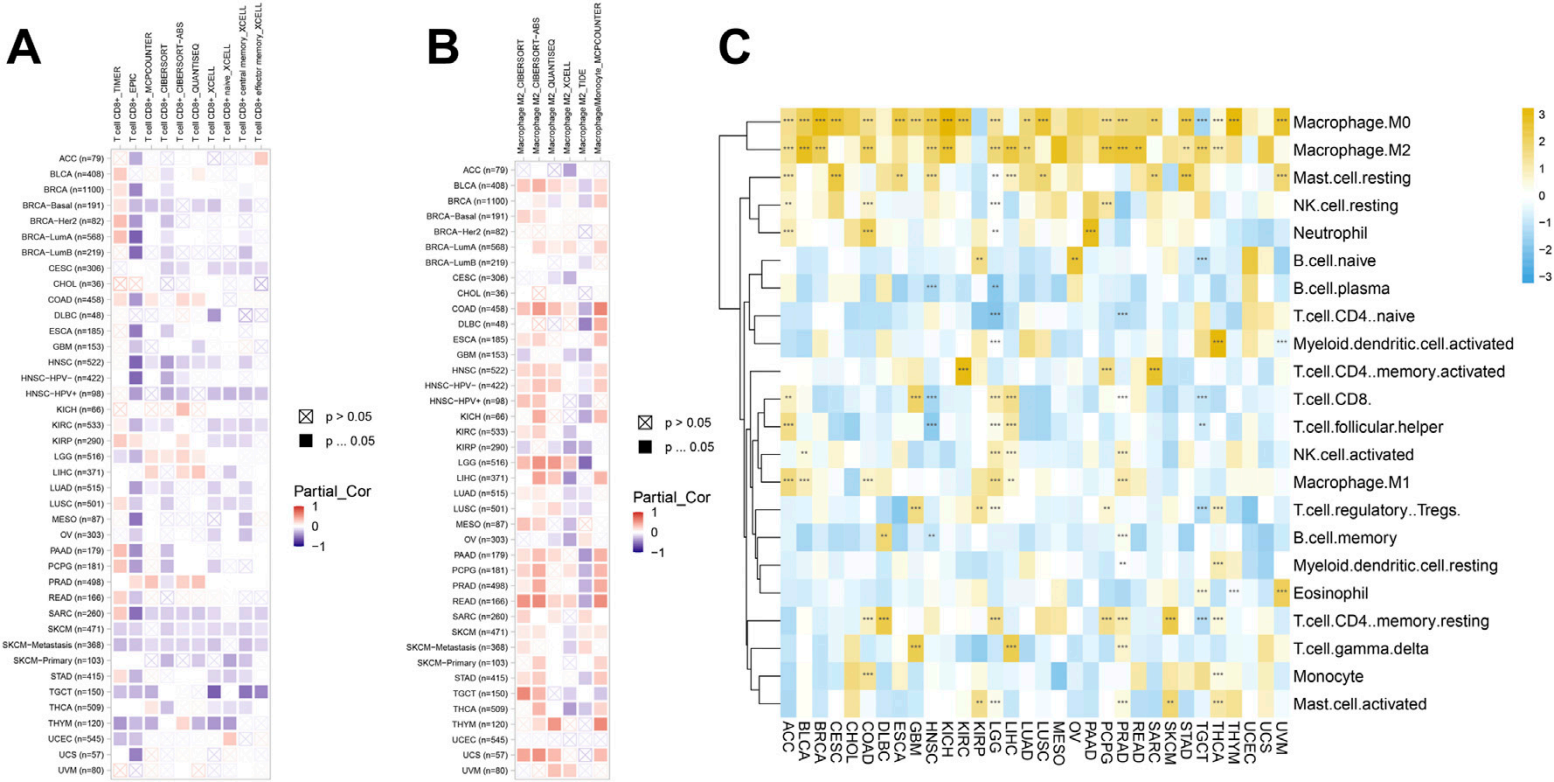
Immune cell infiltration analyses. **(A)** Association between SERPINH1 and infiltration level of CD8+ T cells. **(B)** Association between SERPINH1 and infiltration level of macrophage M2. **(C)** Association between SERPINH1 and infiltration level of indicated immune cells. *p < 0.05; **p < 0.01; ***p < 0.001, and ****p < 0.0001.

Immune cells were closely related to metabolism, development, and occurrence of tumor. So, we further studied the relationship between SERPINH1 expression and immune cell infiltration level using TIMER2 online database. The results of the infiltration level demonstrated that SERPINH1 was negatively correlated with CD8^+^ T cells but positively associated with M2 macrophages through different algorithms in pan-cancer (Figures 10A,B). It suggested that SERPINH1 may inhibit the function of immune cells by inhibiting CD8^+^ T cells and promoting the infiltration of M2 macrophages to promote the progression of tumors.

To further explore the connection between SERPINH1 and the infiltration of various immune cells in pan-cancer, we analyzed the 22 immune cells by using the CIBERSORT method. This result verified that SERPINH1 was negatively correlated with CD8^+^ T cells, while positively correlated with M2 macrophage once again (Figure 10C). That means SERPINH1 may play a risk factor in most human cancers.

### Correlation between SERPINH1 expression and immune regulation–related genes

The relationship between the expression of SERPINH1 and immune score, stromal score, and the estimate score is shown in Supplementary Figures S4–S6. The results revealed that SERPINH1 expression had a positive connection to immune score in the ACC, CESC, DLBC, ESCA, GBM, KICH, LUAC, LUSC, and STAD (Supplementary Figure S4), while it had a positive association with stromal score in the CESC, HNSC, KICH, KIRP, MESO, OV, PAAD, SKCM, TGCT, UCEC, and UCS (Supplementary Figure S5). The same was found in the estimate score, that is, it was positively related to DLBC, ESCA, GBM, KICH, LUAD, LUSC, and STAD (Supplementary Figure S6).

**FIGURE 11:**
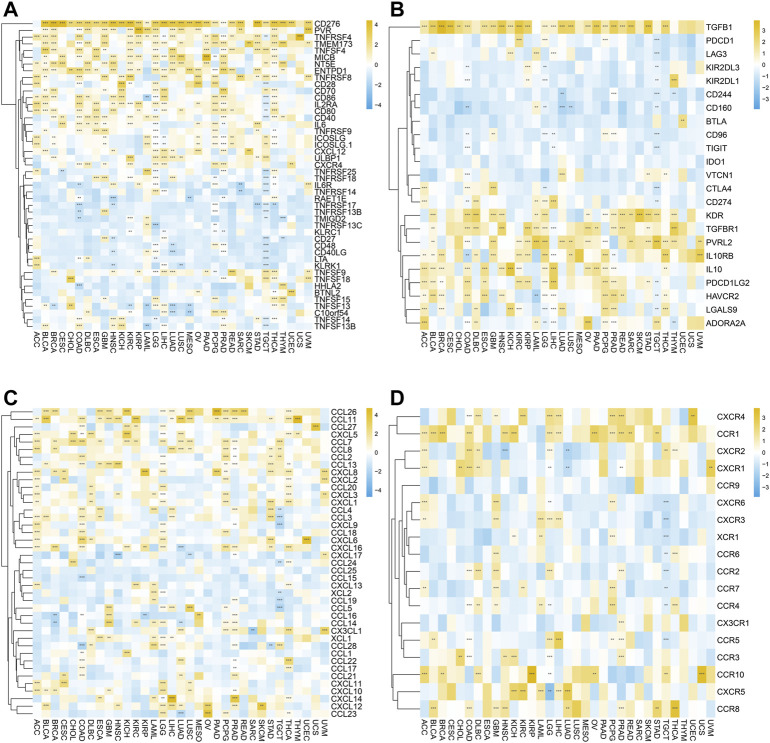
Correlation between SERPINH1 expression and immune regulation–related genes as well as chemokines. These heatmaps, respectively, indicate the correlation between the expression of SERPINH1 and immune activation genes **(A)**, immunosuppressive state-related genes **(B)**, chemokine genes **(C)**, and chemokine receptor genes **(D)**. *p < 0.05; **p < 0.01; ***p < 0.001, and ****p < 0.0001.

Moreover, we further investigated the relationship between SERPINH1 expression and immune regulation–related genes as well as chemokines. As shown in Figure 11A, the correlation analysis of 46 immune-stimulating genes demonstrated that SERPINH1 expression was highly associated with CD276 in most cancers. Also, SERPINH1 expression was also positively associated with PVR, TNFRSF4, and TMEM173. The result of Figure 11B showed that SERPINH1 expression was positively related to the following immunosuppressive genes in pan-cancer, including TGFB1, KDR, TGFBR1, PVRL2, IL 10RB, and IL 10. Moreover, SERPINH1 expression was positively associated with many kinds of chemokines, especially CCL 26 and CCL 11 (Figure 11C). In addition, we can find that its expression positively related to multiple chemokines receptors in pan-cancer, such as CXCR4, CCR1, and CCR10 (Figure 11D).

The abovementioned results suggested that SERPINH1 may participate in immunoregulation and chemotactic recruitment.

### SERPINH1 expression is associated with immune checkpoint genes in human cancers

**FIGURE 12:**
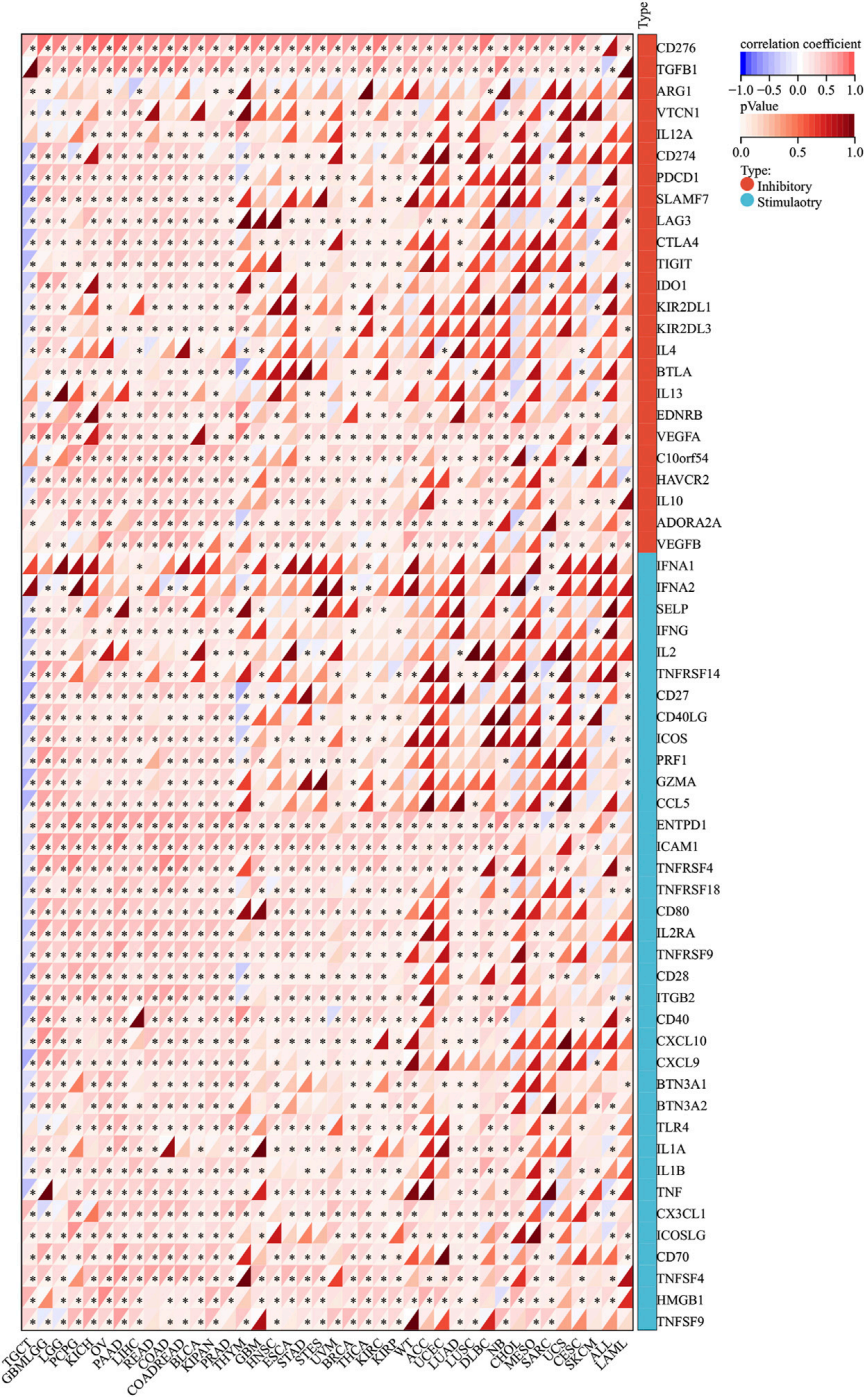
Relationship between SERPINH1 expression and pan-cancer immune checkpoint genes. *p < 0.05.

Normally, the immune system can recognize and eliminate tumor cells in the tumor microenvironment. However, tumor cells have adopted diverse strategies to escape the surveillance of the immune system and survive. Tumor immunotherapy is a treatment method to control and eliminate tumors, and monoclonal antibody immune checkpoint inhibitors are one of them. Therefore, we analyzed the correlation between SERPINH1 expression and 60 immune checkpoint (ICP) genes in this study. The result showed that SERPINH1 had a positive relationship with GBMLGG, LGG, PCPG (pheochromocytoma and paraganglioma), KICH, OV, PAAD (pancreatic adenocarcinoma), LIHC, READ, COAD, COADREAD, BLCA, KIPAN, PRAD, BRCA, THCA (Thyroid carcinoma), KIRC, and KIRP among 60 ICP genes, while it had a negative correlation with TGCT. Notably, CD276, PGFBI, ENTPDI, and ICAM1 were highly expressed in most of the human cancers (Figure 12). This result suggested that SERPINH1 might regulate the activity of these CIP genes in various pathways. That means SERPINH1 might become a potential immunotherapy target in some carcinomas.

### SERPINH1 association between expression and ICB response

The correlation between SERPINH1 expression and immune checkpoint blockade (ICB) response was validated by analyzing data from GSE78220 and IMvigor210. Patients with lower SERPINH1 expression were more responsive to immunotherapy (Supplementary Figures S7A,B). Also, significant differences were found in SERPINH1 expression in patients with different response. This suggested that patients with low SERPINH1 expression could be a potentially beneficial population for ICB treatment.

### Single-cell transcriptional analysis of SERPINH1 in the KIRC tumor microenvironment

**FIGURE 13:**
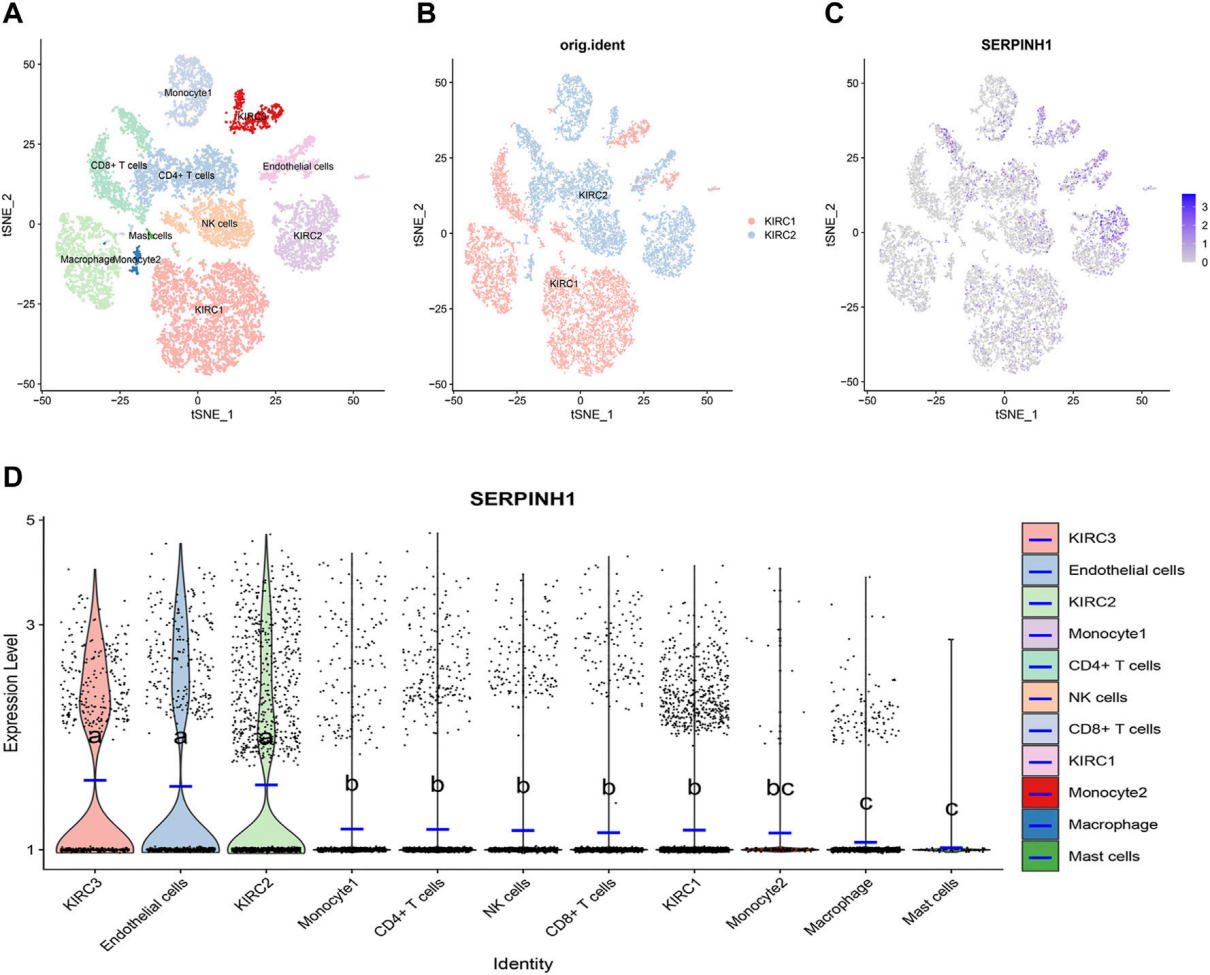
Single-cell transcriptomic Atlas of KIRC. **(A)** tSEN plot representation of KIRC samples with 11 distinct cell types. **(B)** tSEN plot representation of KIRC from two different samples. **(C)** tSEN plot representation of SERPINH1 in different cell types. **(D)** Comparison of SERPINH1 expression in different KIRC tumor microenvironment cells. The blue horizontal line on the violin plot indicates the median SERPINH1 expression. The letters at the top indicate a statistical difference between cells for two comparisons. Different letters indicate that the difference is statistically significant.

ScRNA-seq was performed on two KIRC samples. Subsequently, information about 13124 high-quality single-cell transcriptomes was used for the analysis after conducting QC using Seurat. Cell clustering analysis based on the tSNE algorithm showed that the cells could be classified into 11 clusters, namely, KIRC1, KIRC2, KIRC3, monocyte1, monocyte2, macrophage, mast cells, endothelial cells, NK cells, CD4^+^ T cells, and CD8^+^ T cells (Figure 13A). In addition, it was observed that tumor cells from two different sources of KIRC samples had the same cluster (KIRC3) and unique clusters (KIRC1 and KIRC2) (Figure 13B). Thus, the results suggested heterogeneity of KIRC cell types. We explored the expression of SERPINH1 in KIRC tumor microenvironment cells and compared the SERPINH1 expression across cell types (Figure 13C). Interestingly, we found a significant difference in the SERPINH1 of the abovementioned 11 types of cells (Figure 13D). Moreover, SERPINH1 expression was highest in KIRC cells and endothelial cells but lowest in macrophages and mast cells. The expression of SERPINH1 was significantly higher in tumor cells and endothelial cells than in immune cells (*p* < 0.05). Therefore, targeting SERPINH1 may be more lethal to tumor cells. We used CancerSEA to explore the function of SERPINH1 at the single-cell level. We found that SERPINH1 was significantly associated with invasion, DNA repair, inflammation, metastasis, and angiogenesis in GBM. In addition, SERPINH1 was strongly associated with EMT and metastasis in most tumors (Supplementary Figure S8).

## Discussion

Nowadays, most tumors cannot be cured surgically, especially at an advanced stage and metastatic tumors. Previous studies have found that tumors are closely related to the human immune microenvironment. Therefore, the treatment of tumors by immunotherapy has become the main research focus at present. SERPINH1, also known as HSP47, is a collagen-specific molecular chaperone located in the endoplasmic reticulum (Ito and Nagata, 2017). In recent years, SEEPINH1 had been researched upon in many cancers, including gastric (GC) and CRC. Research showed that SERPINH1 could be a serum marker in diagnosis and prognosis assessment of GC (Liu et al., 2021) and potentially be the noninvasive biomarker reflecting the adenoma and carcinoma, especially CRC (Zhang et al., 2021). But, the relationship between SERPINH1 and immune-related therapy is not clear in pan-cancer. So, we analyzed the SERPINH1 expression in pan-cancer to find out its value in immunotherapy.

Our results indicated that SERPINH1 expression was highly expressed in most human tumors using the Oncomine database. In addition, amplification and missense mutations are the main genetic types of SERPINH1 genetic alteration. We also found that SERPINH1 expression has a positive correlation with tumor stage in pan-cancer according to our research. The result of GSEA of SEIPINH1 also revealed that SERPINH1 overexpression was related to malignant progression of tumors. For the protein information of SERPINH1, the result demonstrated that SERPINH1 protein level was higher in tissues of colon, liver, and lung cancer. In the PPI network, we found that SERPINH1 had a close relationship with many collagens. A previous study showed that SERPINH1 could promote the maturation of procollagen, which is closely related to tumor metastasis (Gyorffy et al., 2012; Xia et al., 2015).

The Kaplan–Meier analysis of OS, DSS, and PFI also represented that SERPINH1 is a risk factor for patients in many kinds of cancer. Some recent studies have found that SERPINH1 is overexpressed in KICR (Qi et al., 2018), ESCA (Kwon et al., 2009), GBMLGG (Zhao et al., 2014), PAAD (Duarte and Bonatto, 2018), HNSC (Lee et al., 2011), and CRC (Mori et al., 2017). Our results were consistent with those studies that had been researched before. These results indicated that SERPINH1 higher expression was significantly associated with worse survival duration. Considering the probability of Kaplan–Meier analysis was limited, we added the univariate Cox regression analysis. The results of its OS, DSS, and PFI analyses further confirmed that SERPINH1 is a worse factor in various cancers. In addition, research had proved that knocking down SERPINH1 could observably inhibit the invasion, migration, and proliferation of tumor cells (Yamamoto et al., 2013). That means SERPINH1 may be significantly related to poor prognosis in pan-cancer.

Through the immune cell infiltration analysis, we discovered that SERPINH1 was negatively correlated with CD8^+^ T cells but positively associated with M2 macrophages. CD8^+^ T cells can kill tumor cells with cytotoxic molecules such as granzymes and perforin and form an important defense system of antitumor immunity with natural killer cells. Previous studies showed that the infiltration of CD8^+^ T cells in the tumor microenvironment had a notable relationship with better prognosis in many malignant cancers, such as breast, brain, lung, and colorectal cancer (Fridman et al., 2012; Reiser and Banerjee, 2016). However, M2 macrophage can secrete inhibitory cytokines, such as IL-10 or TGF-B, to downregulate immune response and promote the development of tumor. Therefore, SERPINH1 might influence the infiltration of CD8^+^ T cells and M2 macrophages by affecting the tumor microenvironment–related immune pathways to promote tumor development. Previous studies have known that macrophages can produce different species of collagen (Schnoor et al., 2008). In addition, well-organized and interconnected collagen network can cut down the amount of penetration of drug in solid cancers. A study showed that drugs were isolated by binding with extracellular matrix components to inhibit drug permeation to deeper areas of carcinomas (Berk et al., 1997). Another explanation is that the final assembly of macrophage-related collagen leads to the stress of endoplasmic reticulum and activation of macrophage (Ma and Hendershot, 2004). But the relevant immune mechanism is not clear yet and needs further study.

For the analysis of 24 immune-inhibiting genes, we found that SERPINH1 expression was highly associated with CD276, TGFBI, VEGFA, HAVCR2, IL10, and ADORA2A in most cancers. SERPINH1 expression was positively related toENTPD1, ICAM1, and TNFRSF4 in 36 immunoactivating genes. Some research studies had attested that high expression of CD276 had been confirmed to be probably related to the immune escape of tumor cells, and its overexpression in tumor cells had a strong interference and metastasis ability. It is a physiological barrier for tumors to resist immune regulation (Wang et al., 2021a). This suggested that SERPINH1 can regulate the immunity of organisms by changing the immune regulation–related genes to affect tumors. Our study also revealed that SERPINH1 had a close connection with many chemokines, especially CCL 26 and CCL 11. In addition, SERPINH1 also related to their receptors in pan-cancer, such as CXCR4, CCR1, and CCR10. These results further confirmed that SERPINH1 has a significant relationship with the immune system.

The association of immune checkpoint genes in human cancers and SERPINH1 showed that SERPINH1 had a positive relationship with GBMLGG, LGG, PCPG, KICH, OV, PAAD, LIHC, READ, COAD, COADREAD, BLCA, KIPAN, PRAD, BRCA, THCA, KIRC, and KIRP among 60 immune check point genes, while negative correlation with TGCT in 60 ICP gene analysis. These results demonstrated the role of SERPINH1 potential target for tumor immunotherapy. From the results of the correlation between SERPINH1 expression and ICB response, we found that SERPINH1 underexpression was more responsive to immunotherapy and higher expression was insensitive. This suggested that patients with SERPINH1 underexpression could be a potential beneficiary group for ICB treatment. But, the relevant mechanism of immune pathways between tumors and SERPINH1 is not very clear now. Also, we need more experiments to test the curative effect of SERPINH1 target therapy in clinical trials.

Pan-cancer analysis is a comprehensive analysis that encompasses a wide range of analytical methods and components. Although Wang *et al* have provided a preliminary exploration of the role of SERPINH1 in pan-cancer (Wang et al., 2021b), our study was innovative in the following ways. Our study had more rational and innovative data sources. As is well known, the TCGA contains 33 tumors from different data collection points, using different data processing processes. A batch effect was found between the different data (Rasnic et al., 2019). Without standardization of the data such as de-batching, the results of subsequent analyses may be affected. The final result of the TCGA project, the most comprehensive cross-cancer genomic analysis to date (Pan-Cancer Atlas) of over 11,000 samples from 33 cancers over more than a decade, the Pan-Cancer Atlas uses a standardized process for processing different data, removing batch effects, and making the different data more comparable and increasing confidence in the results (Hoadley et al., 2018). Our analyses are derived from the standardized data generated by the TCGA Pan-Cancer project, making the results more reliable. Our study used innovative analysis methods. Taking enrichment analysis as an example, we used GSEA to explore the function of SERPINH1. Conventional enrichment analysis focuses on a list of genes, concentrating on a few selected genes, which can lead to omission of critical information due to poor screening parameters, for example, some genes that are not significantly differentially expressed but are biologically important (Geistlinger et al., 2021). The advantage of GSEA is that, instead of artificially selecting a list of genes, the expression of all genes can be analyzed directly and a nonsignificant but consistent set of differentially expressed genes can be detected. The advantages of GSEA have been increasingly recognized and are becoming the mainstream method for gene enrichment analysis (Croken et al., 2014). Our study was the first assessment of the relationship between SERPINH1 expression and ICB response using multiple immunotherapy cohorts. For the first time, single-cell transcriptome sequencing data were used to assess the expression of SERPINH1 in different cells of the tumor microenvironment. Ours was the first single-cell-based approach to explore the molecular function of SERPINH1.

Despite the systematic analysis of SERPINH1, there are inevitable limitations in this study. First, the exact significance of SERPINH1 expression in various cancers and the exact implication of immunoregulation in these cancers remain incomplete. Second, direct clinical trials are further required to clarify the capacity of SERPINH1 in regulating the immune process.

## Conclusion

SERINH1 may act as a risk factor in tumor development and is related to poor prognosis in pan-cancer. SERPINH1 expression may contribute to the regulation of immune cells and its metabolites, such as CD8^+^ T cells, M2 macrophages, and CD276. Therefore, SERPINH1 is likely to be a potential target in immunotherapy or become a prognosis biomarker in pan-cancer.

## Data Availability

The original contributions presented in the study are included in the article/Supplementary Material; further inquiries can be directed to the corresponding authors.
