# Supplementary figures and images for: Prognostic and immunological role of SERPINH1 in pan-cancer
Source: Front Genet. 2022 Aug 29;13:900495. doi: 10.3389/fgene.2022.900495 (PMC9465257; doi:10.3389/fgene.2022.900495)

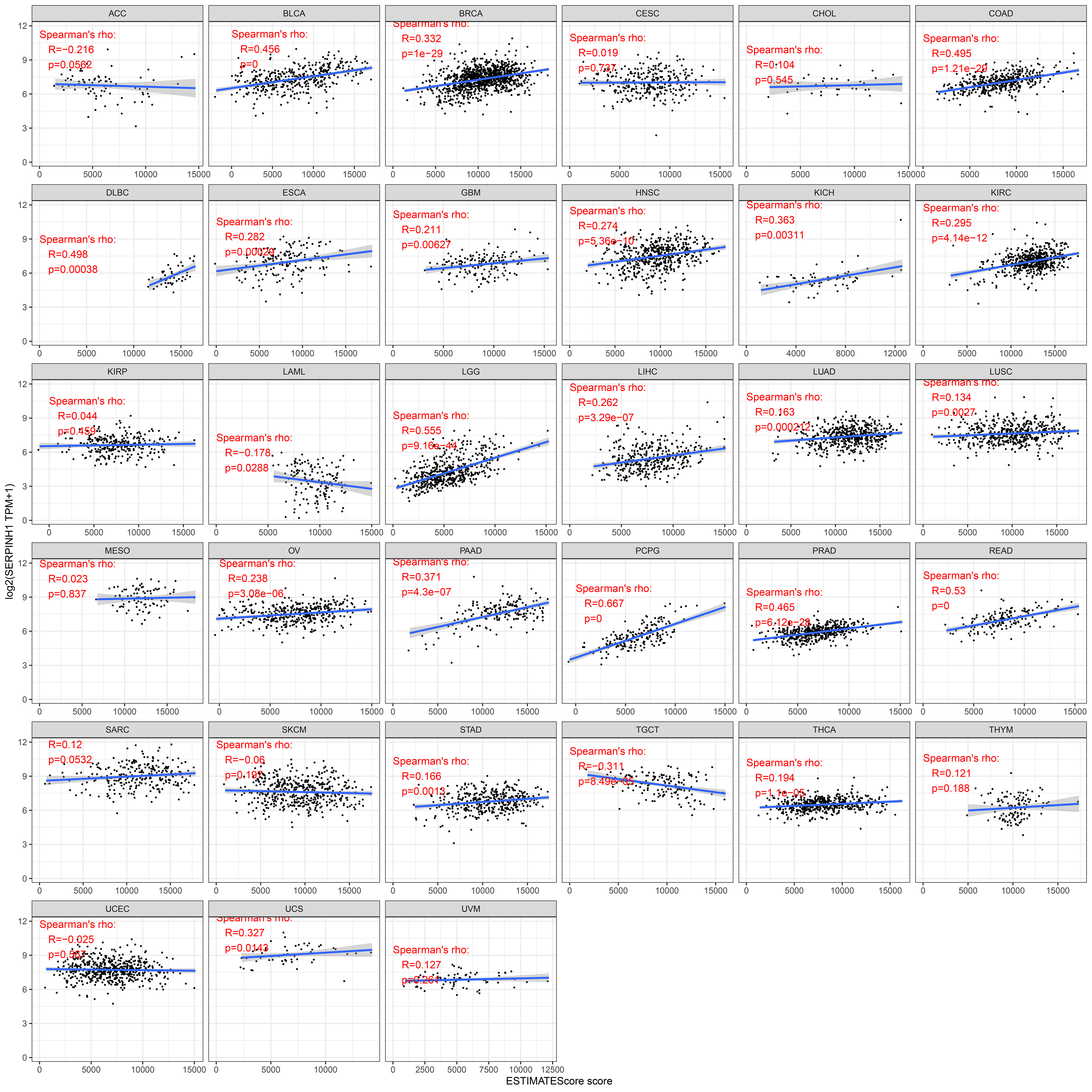

Supplement: Supplementary file 1 [file Image6.TIF]

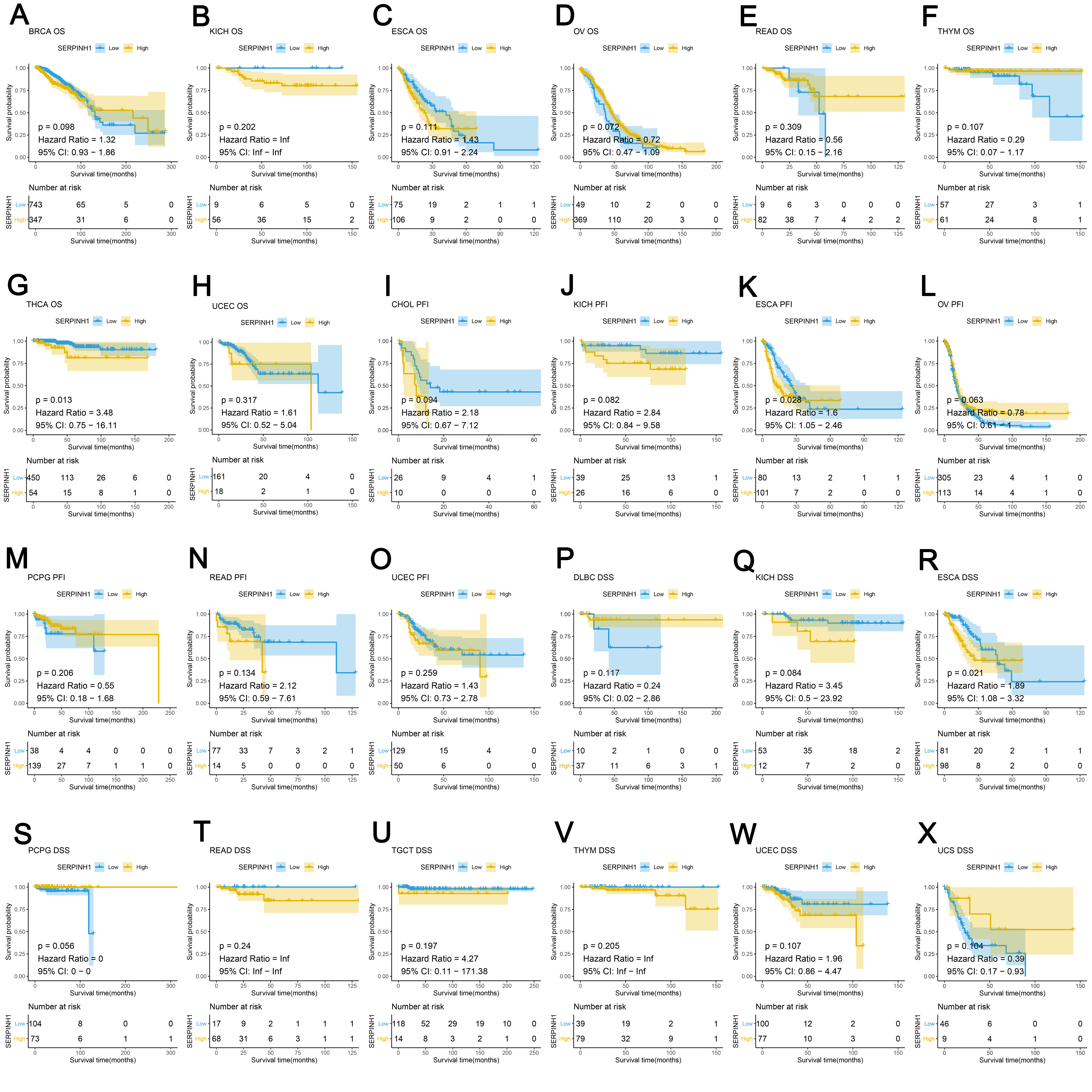

Supplement: Supplementary file 2 [file Image3.TIF]

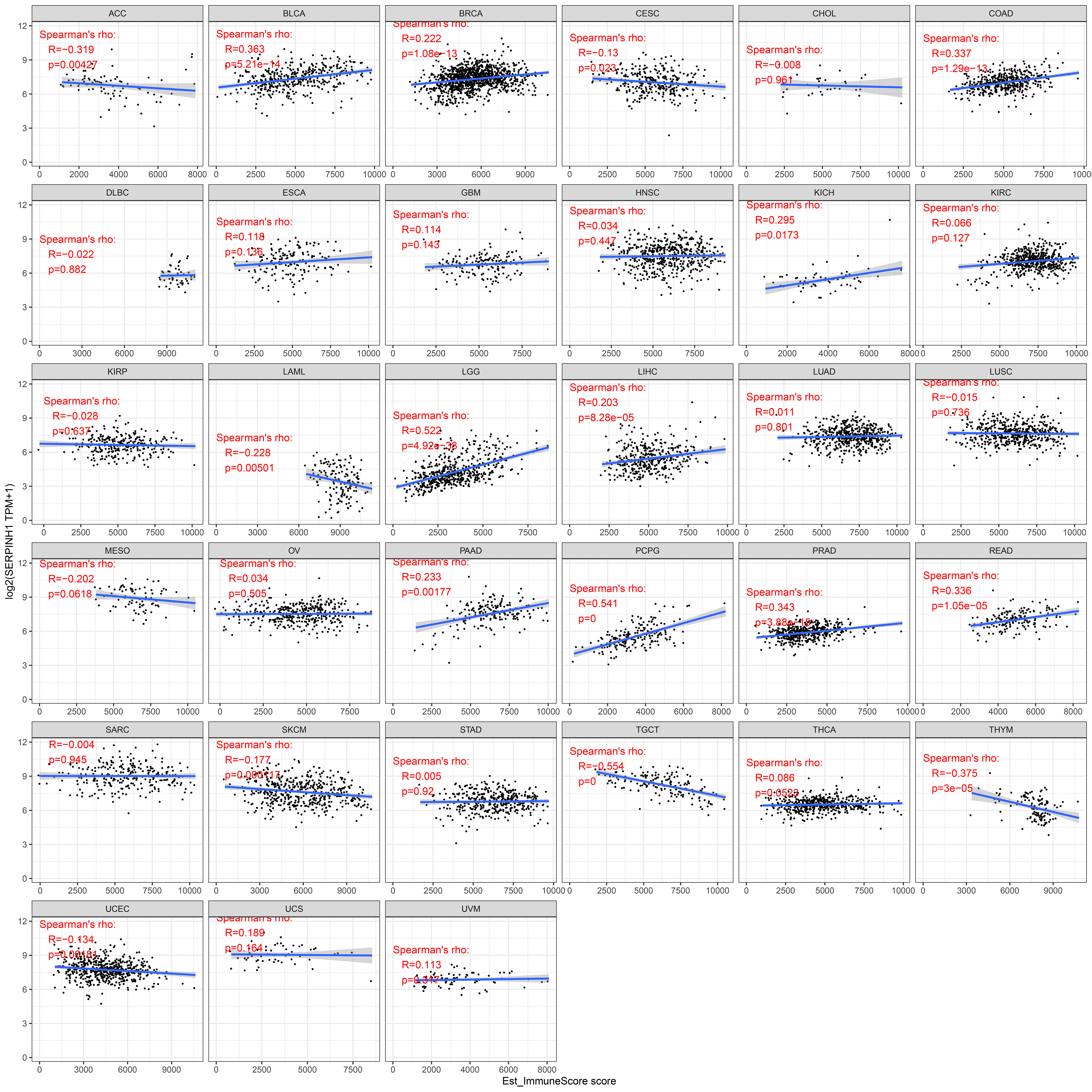

Supplement: Supplementary file 3 [file Image4.TIF]

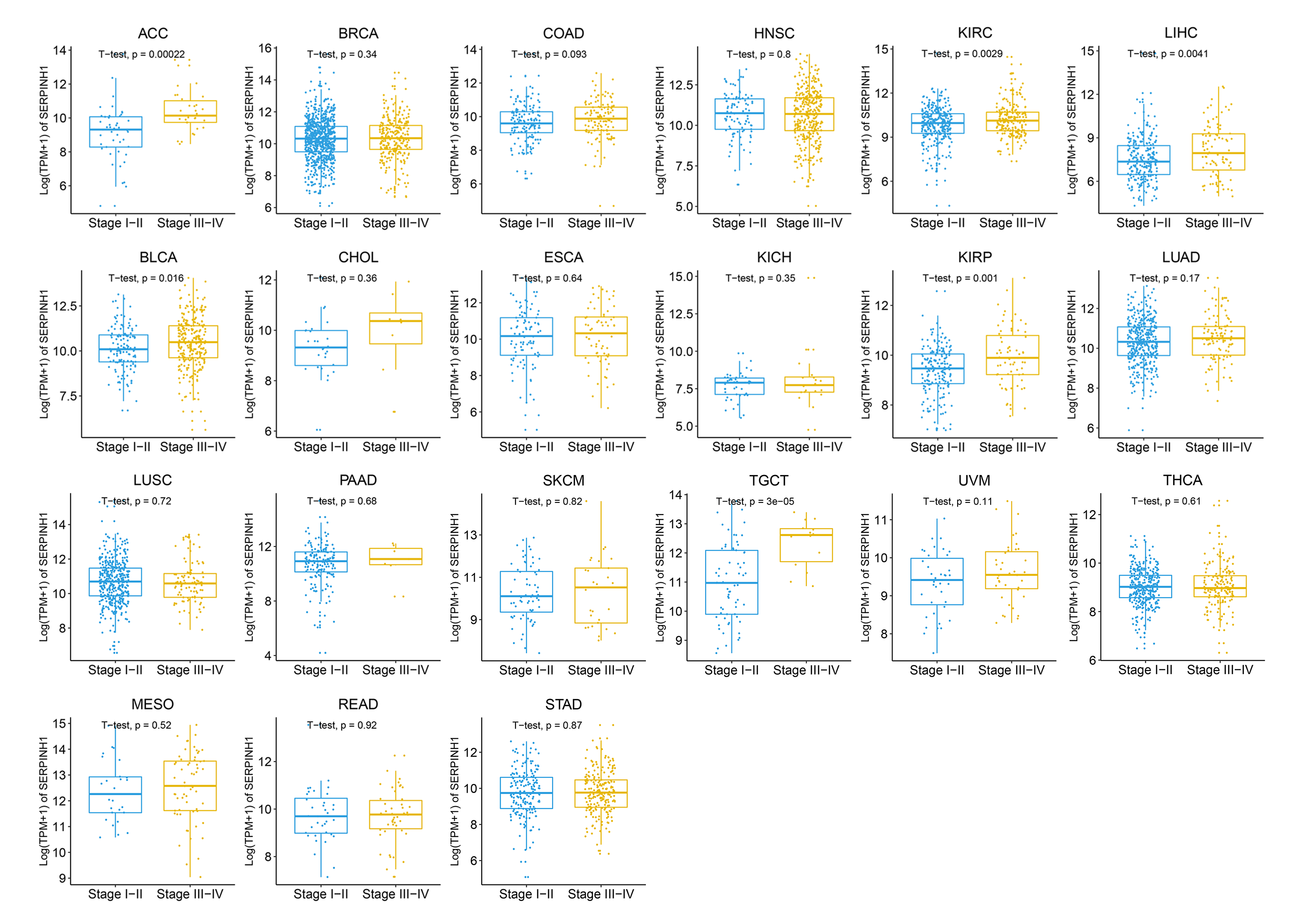

Supplement: Supplementary file 4 [file Image2.TIF]

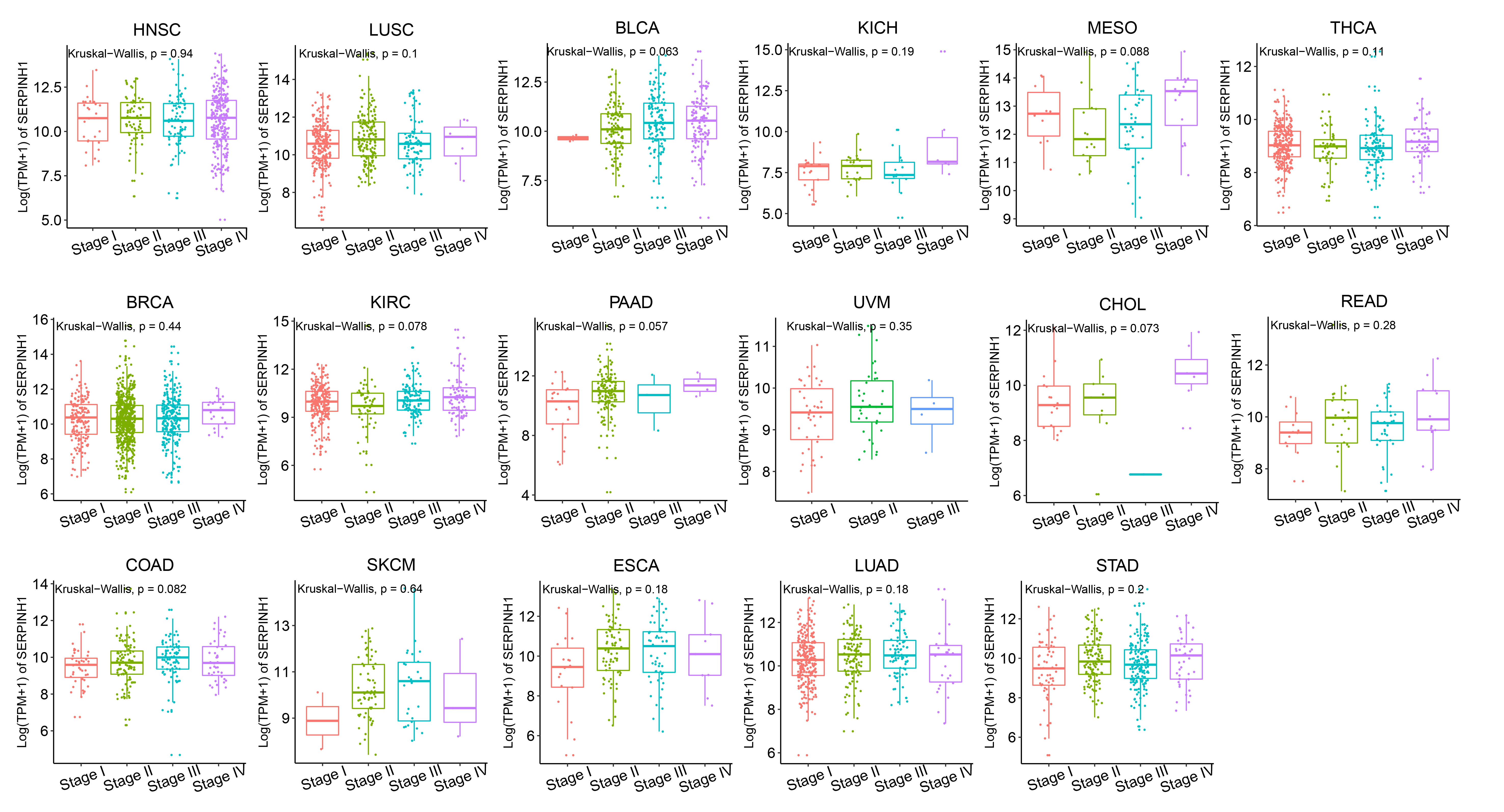

Supplement: Supplementary file 5 [file Image1.TIF]

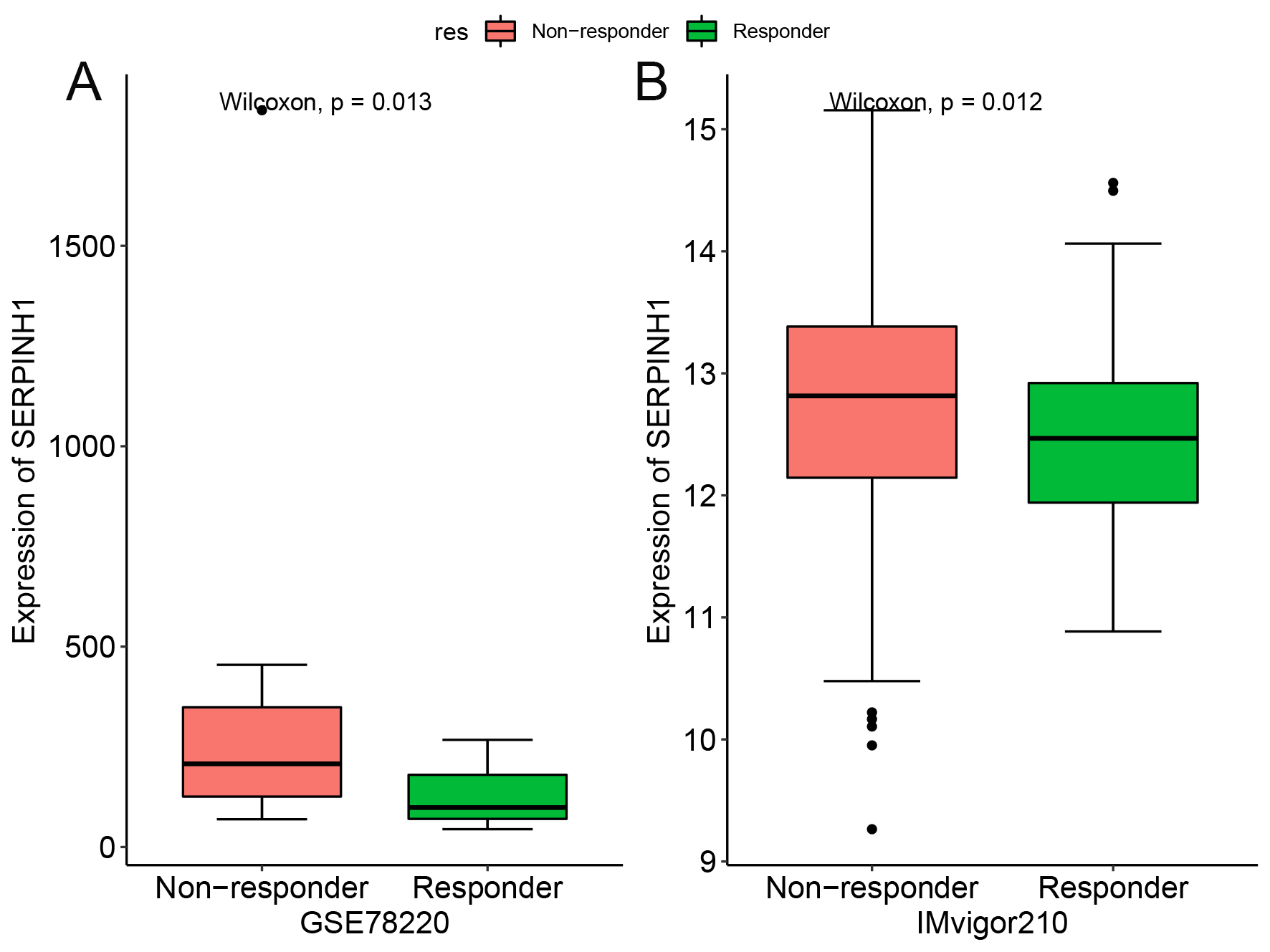

Supplement: Supplementary file 6 [file Image7.TIF]

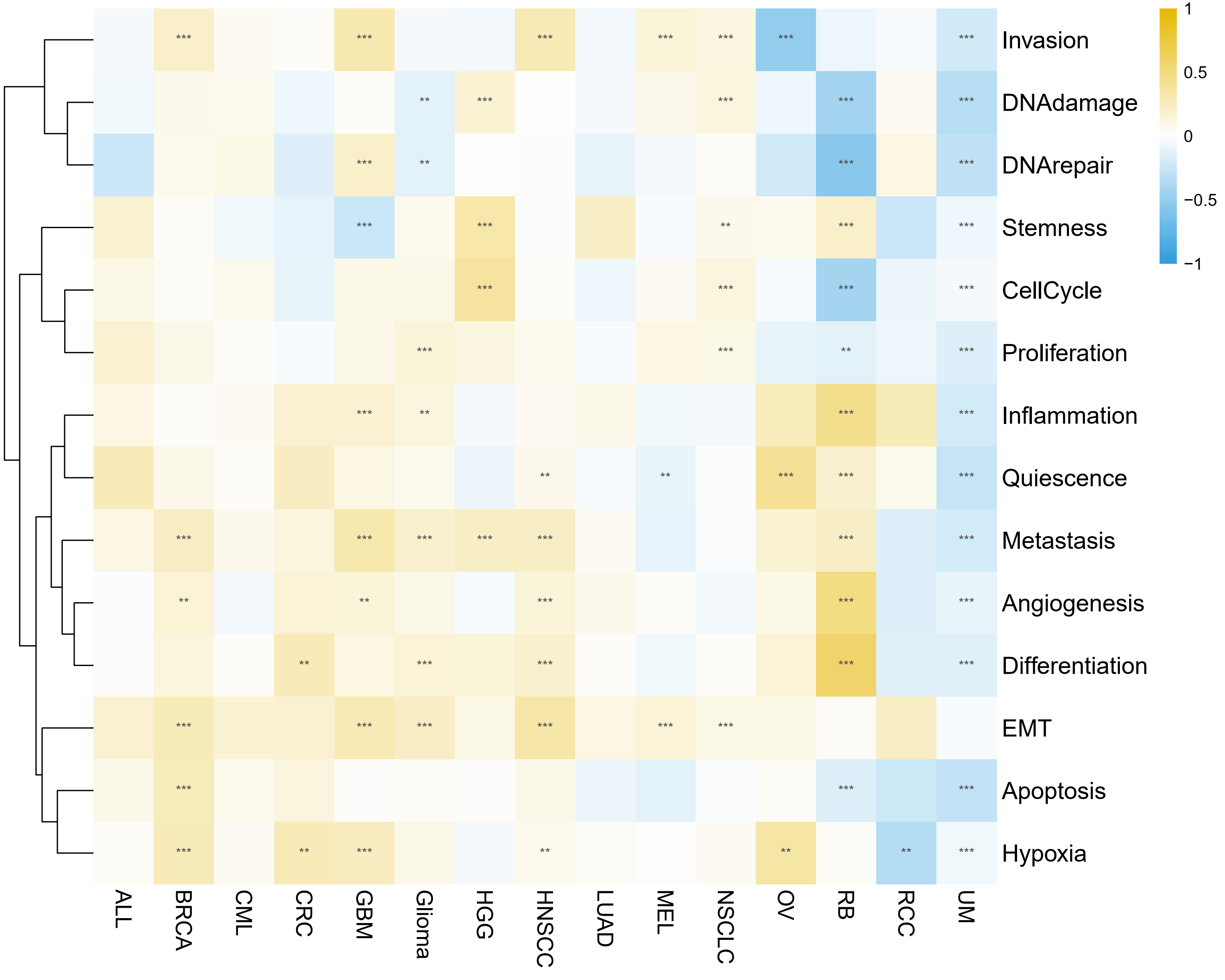

Supplement: Supplementary file 9 [file Image8.TIF]

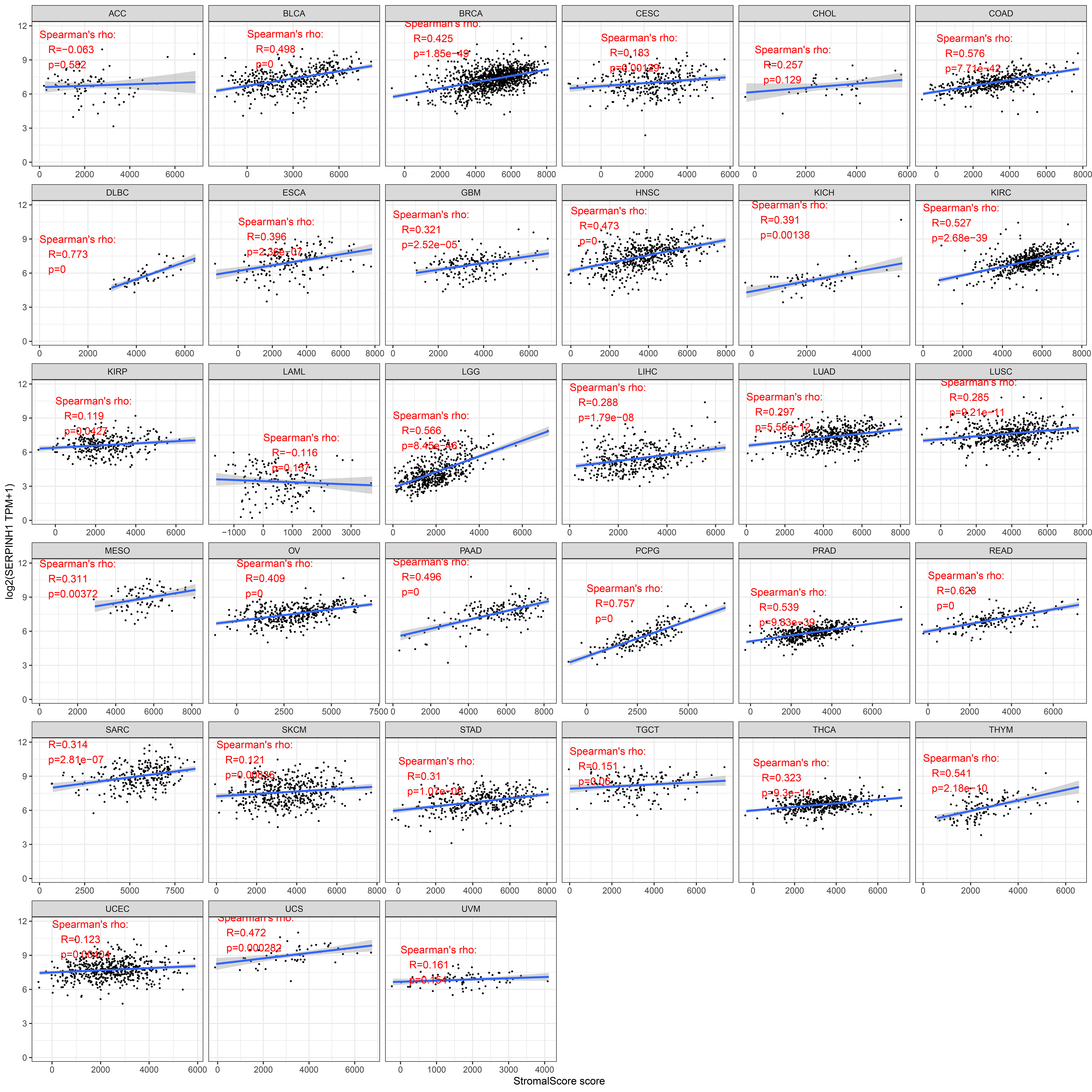

Supplement: Supplementary file 10 [file Image5.TIF]
