# Supplementary material for: Prognostic and immunological role of SERPINH1 in pan-cancer
Source: Front Genet. 2022 Aug 29;13:900495. doi: 10.3389/fgene.2022.900495 (PMC9465257; doi:10.3389/fgene.2022.900495)
Supplement: Supplementary file 7 [file Table2.DOCX]

Supplementary Table 1: Correspondence table of Chinese and English names and abbreviations of TCGA cancer

| **Abbreviation** | **Full name** | **Abbreviation** | **Full name** |
| --- | --- | --- | --- |
| ACC | Adrenocortical carcinoma | BLCA | Bladder Urothelial Carcinoma |
| BRCA | Breast invasive carcinoma | CESC | Cervical squamous cell carcinoma and endocervical adenocarcinoma |
| CHOL | Cholangiocarcinoma | COAD | Colon adenocarcinoma |
| COADREAD | Colon adenocarcinoma/Rectum adenocarcinoma Esophageal carcinoma | DLBC | Lymphoid Neoplasm Diffuse Large B-cell Lymphoma |
| ESCA | Esophageal carcinoma | FPPP | FFPE Pilot Phase ll |
| GBM | Glioblastoma multiforme | GBMLGG | Glioma |
| HNSC | Head and Neck squamous cell carcinoma | KICH | Kidney Chromophobe |
| KIRC | Kidney renal clear cell carcinoma | KIRP | Kidney renal papillary cell carcinoma |
| LAML | Acute Myeloid Leukemia | LGG | Brain Lower Grade Glioma |
| LIHC | Liver hepatocellular carcinoma | LUAD | Lung adenocarcinoma |
| LUSC | Lung squamous cell carcinoma | MESo | Mesothelioma |
| OV | Ovarian serous cystadenocarcinoma | PAAD | Pancreatic adenocarcinoma |
| PCPG | Pheochromocytoma and Paraganglioma | PRAD | Prostate adenocarcinoma |
| READ | Rectum adenocarcinoma | SARC | Sarcoma |
| SKCM | Skin Cutaneous Melanoma | STAD | Stomach adenocarcinoma |
| STES | Stomach and Esophageal carcinoma | TGCT | Testicular Germ Cell Tumors |
| THCA | Thyroid carcinoma | THYM | Thymoma |
| UCEC | Uterine Corpus Endometrial Carcinoma | UCS | Uterine Carcinosarcoma |
| UVM | Uveal Melanoma | AML | Acute Myeloid Leukemia |
| CCSK | Clear Cell Sarcoma of the Kidney | NBL | Neuroblastoma |
| OS | Osteosarcoma | RT | Rhabdoid Tumor |
| WT | High-Risk Wilms Tumor |  |  |
