# Supplementary material for: Prognostic and immunological role of SERPINH1 in pan-cancer
Source: Front Genet. 2022 Aug 29;13:900495. doi: 10.3389/fgene.2022.900495 (PMC9465257; doi:10.3389/fgene.2022.900495)
Supplement: Supplementary file 8 [file Table3.DOCX]

| Supplementary Table 2: Hazard ratio and p-value for OS, DSS, and PFI in different cancers. | | | | | | | |
| --- | --- | --- | --- | --- | --- | --- | --- |
| ACC | OS | Hazard Ratio | 7.42 | COAD | OS | Hazard Ratio | 1.99 |
|  |  | 95% CI | 1.92-28.76 |  |  | 95% CI | 1.22-3.24 |
|  |  | p-value | ＜0.001 |  |  | p-value | 0.004 |
|  | PFI | Hazard Ratio | 4.64 |  | PFI | Hazard Ratio | 1.54 |
|  |  | 95% CI | 2.46-8.75 |  |  | 95% CI | 0.99-2.4 |
|  |  | p-value | ＜0.001 |  |  | p-value | 0.047 |
|  | DSS | Hazard Ratio | 5.97 |  | DSS | Hazard Ratio | 2.7 |
|  |  | 95% CI | 2.01-17.72 |  |  | 95% CI | 1.38-5.27 |
|  |  | p-value | ＜0.001 |  |  | p-value | 0.003 |
| BLCA | OS | Hazard Ratio | 1.44 | GBM | OS | Hazard Ratio | 1.75 |
|  |  | 95% CI | 1.05-1.98 |  |  | 95% CI | 1.15-2.67 |
|  |  | p-value | 0.016 |  |  | p-value | 0.016 |
|  | PFI | Hazard Ratio | 2.18 |  | PFI | Hazard Ratio | 1.81 |
|  |  | 95% CI | 1.28-3.72 |  |  | 95% CI | 1.25-2.64 |
|  |  | p-value | ＜0.001 |  |  | p-value | ＜0.001 |
|  | DSS | Hazard Ratio | 1.84 |  | DSS | Hazard Ratio | 1.71 |
|  |  | 95% CI | 1.15-2.97 |  |  | 95% CI | 1.14-2.56 |
|  |  | p-value | 0.002 |  |  | p-value | 0.004 |
| CESC | OS | Hazard Ratio | 2.55 | HNSC | OS | Hazard Ratio | 1.62 |
|  |  | 95% CI | 1.51-4.33 |  |  | 95% CI | 1.23-2.14 |
|  |  | p-value | ＜0.001 |  |  | p-value | ＜0.001 |
|  | PFI | Hazard Ratio | 2.76 |  | PFI | Hazard Ratio | 1.66 |
|  |  | 95% CI | 1.72-4.41 |  |  | 95% CI | 1.22-2.27 |
|  |  | p-value | ＜0.001 |  |  | p-value | ＜0.001 |
|  | DSS | Hazard Ratio | 2.74 |  | DSS | Hazard Ratio | 2 |
|  |  | 95% CI | 1.6-4.69 |  |  | 95% CI | 1.37-2.93 |
|  |  | p-value | ＜0.001 |  |  | p-value | ＜0.001 |
| CHOL | OS | Hazard Ratio | 3.5 | KIRC | OS | Hazard Ratio | 2.25 |
|  |  | 95% CI | 0.97-12.59 |  |  | 95% CI | 1.3-3.92 |
|  |  | p-value | 0.005 |  |  | p-value | ＜0.001 |
| BRCA | PFI | Hazard Ratio | 2.42 |  | PFI | Hazard Ratio | 2.27 |
|  |  | 95% CI | 1.63-3.59 |  |  | 95% CI | 1.53-3.35 |
|  |  | p-value | ＜0.001 |  |  | p-value | ＜0.001 |
|  | DSS | Hazard Ratio | 2.1 |  | DSS | Hazard Ratio | 2.34 |
|  |  | 95% CI | 1.36-3.23 |  |  | 95% CI | 1.47-3.73 |
|  |  | p-value | 0.002 |  |  | p-value | ＜0.001 |
| KIRP | OS | Hazard Ratio | 7.39 | LUSC | OS | Hazard Ratio | 1.41 |
|  |  | 95% CI | 1.99-27.4 |  |  | 95% CI | 1.02-1.95 |
|  |  | p-value | ＜0.001 |  |  | p-value | 0.023 |
|  | PFI | Hazard Ratio | -- |  | PFI | Hazard Ratio | 2.42 |
|  |  | 95% CI | -- |  |  | 95% CI | 1.43-4.07 |
|  |  | p-value | -- |  |  | p-value | 0.019 |
|  | DSS | Hazard Ratio | 9.81 |  | DSS | Hazard Ratio | 1.62 |
|  |  | 95% CI | 2.11-45.62 |  |  | 95% CI | 1.05-2.51 |
|  |  | p-value | ＜0.001 |  |  | p-value | 0.023 |
| LGG | OS | Hazard Ratio | 3.87 | MESO | OS | Hazard Ratio | 3 |
|  |  | 95% CI | 2.38-6.31 |  |  | 95% CI | 1.9-4.76 |
|  |  | p-value | ＜0.001 |  |  | p-value | ＜0.001 |
|  | PFI | Hazard Ratio | 3.15 |  | PFI | Hazard Ratio | 3.45 |
|  |  | 95% CI | 2.11-4.7 |  |  | 95% CI | 2.06-5.77 |
|  |  | p-value | ＜0.001 |  |  | p-value | ＜0.001 |
|  | DSS | Hazard Ratio | 4.51 |  | DSS | Hazard Ratio | 4.88 |
|  |  | 95% CI | 2.45-8.31 |  |  | 95% CI | 2.65-8.97 |
|  |  | p-value | ＜0.001 |  |  | p-value | ＜0.001 |

| LIHC | OS | Hazard Ratio | 2.45 | STAD | OS | Hazard Ratio | 1.65 |
| --- | --- | --- | --- | --- | --- | --- | --- |
|  |  | 95% CI | 1.22-4.91 |  |  | 95% CI | 1.02-2.66 |
|  |  | p-value | ＜0.001 |  |  | p-value | 0.013 |
|  | PFI | Hazard Ratio | 1.58 |  | PFI | Hazard Ratio | 1.99 |
|  |  | 95% CI | 1.08-2.32 |  |  | 95% CI | 1.18-3.37 |
|  |  | p-value | 0.007 |  |  | p-value | ＜0.001 |
|  | DSS | Hazard Ratio | 1.78 |  | DSS | Hazard Ratio | 2.3 |
|  |  | 95% CI | 1.14-2.77 |  |  | 95% CI | 1.25-4.26 |
|  |  | p-value | 0.013 |  |  | p-value | ＜0.001 |
| LUAD | OS | Hazard Ratio | 1.88 | THCA | OS | Hazard Ratio | 3.48 |
|  |  | 95% CI | 1.29-2.75 |  |  | 95% CI | 0.75-16.11 |
|  |  | p-value | ＜0.001 |  |  | p-value | 0.013 |
|  | PFI | Hazard Ratio | 1.76 |  | PFI | Hazard Ratio | 0.53 |
|  |  | 95% CI | 1.23-2.52 |  |  | 95% CI | 0.26-1.05 |
|  |  | p-value | 0.011 |  |  | p-value | 0.03 |
|  | DSS | Hazard Ratio | 2.14 |  | DSS | Hazard Ratio | 6.37 |
|  |  | 95% CI | 1.31-3.49 |  |  | 95% CI | 0.57-71.1 |
|  |  | p-value | 0.018 |  |  | p-value | 0.005 |
| PAAD | OS | Hazard Ratio | 3.48 | UVM | OS | Hazard Ratio | 5.04 |
|  |  | 95% CI | 2.02-6.02 |  |  | 95% CI | 0.97-26.16 |
|  |  | p-value | 0.003 |  |  | p-value | ＜0.001 |
|  | PFI | Hazard Ratio | 2.57 |  | PFI | Hazard Ratio | 4.62 |
|  |  | 95% CI | 1.63-4.05 |  |  | 95% CI | 1.59-13.45 |
|  |  | p-value | 0.001 |  |  | p-value | ＜0.001 |
|  | DSS | Hazard Ratio | 3.25 |  | DSS | Hazard Ratio | 5.56 |
|  |  | 95% CI | 1.88-5.62 |  |  | 95% CI | 1.02-30.42 |
|  |  | p-value | 0.003 |  |  | p-value | ＜0.001 |
| PRAD | OS | Hazard Ratio | 3.91 | DLBC | OS | Hazard Ratio | 0.2 |
|  |  | 95% CI | 0.98-15.61 |  |  | 95% CI | 0.02-1.94 |
|  |  | p-value | 0.022 |  |  | p-value | 0.011 |
|  | PFI | Hazard Ratio | 2.62 |  | PFI | Hazard Ratio | 0.2 |
|  |  | 95% CI | 1.46-4.69 |  |  | 95% CI | 0.01-3.81 |
|  |  | p-value | ＜0.001 |  |  | p-value | 0.019 |
|  | DSS | Hazard Ratio | 10.19 |  | DSS | Hazard Ratio | -- |
|  |  | 95% CI | 1.45-71.47 |  |  | 95% CI | -- |
|  |  | p-value | 0.01 |  |  | p-value | -- |
| SARC | OS | Hazard Ratio | 1.8 | UCS | OS | Hazard Ratio | 0.36 |
|  |  | 95% CI | 1.19-2.72 |  |  | 95% CI | 0.16-0.82 |
|  |  | p-value | 0.012 |  |  | p-value | 0.073 |
|  | PFI | Hazard Ratio | 1.7 |  | PFI | Hazard Ratio | 0.47 |
|  |  | 95% CI | 1.2-2.4 |  |  | 95% CI | 0.24-0.9 |
|  |  | p-value | 0.005 |  |  | p-value | 0.035 |
|  | DSS | Hazard Ratio | 1.7 |  | DSS | Hazard Ratio | -- |
|  |  | 95% CI | 1.08-2.68 |  |  | 95% CI | -- |
|  |  | p-value | 0.036 |  |  | p-value | -- |
| SKCM | OS | Hazard Ratio | 3.13 | PCPG | OS | Hazard Ratio | 0 |
|  |  | 95% CI | 1.36-7.19 |  |  | 95% CI | 0-0 |
|  |  | p-value | 0.046 |  |  | p-value | 0.025 |
|  | PFI | Hazard Ratio | 2.05 | THYM | PFI | Hazard Ratio | 0.16 |
|  |  | 95% CI | 1.04-4.06 |  |  | 95% CI | 0.06-0.43 |
|  |  | p-value | 0.026 |  |  | p-value | 0.039 |
|  | DSS | Hazard Ratio | 3.66 | OV | DSS | Hazard Ratio | 0.74 |
|  |  | 95% CI | 1.45-9.25 |  |  | 95% CI | 0.54-1.02 |
|  |  | p-value | 0.003 |  |  | p-value | 0.042 |
